# Development of multivalent mRNA vaccine candidates for seasonal or pandemic influenza

**DOI:** 10.1038/s41541-021-00420-6

**Published:** 2021-12-16

**Authors:** Sudha Chivukula, Timothy Plitnik, Timothy Tibbitts, Shrirang Karve, Anusha Dias, Donghui Zhang, Rebecca Goldman, Hardip Gopani, Asad Khanmohammed, Ashish Sarode, Dustin Cooper, Heesik Yoon, Younghoon Kim, Yanhua Yan, Sophia T. Mundle, Rachel Groppo, Adrien Beauvais, Jinrong Zhang, Natalie G. Anosova, Charles Lai, Lu Li, Gregory Ulinski, Peter Piepenhagen, Joshua DiNapoli, Kirill V. Kalnin, Victoria Landolfi, Ron Swearingen, Tong-Ming Fu, Frank DeRosa, Danilo Casimiro

**Affiliations:** 1 Sanofi Pasteur, Cambridge, MA USA; 2 Emergent Biosolutions, San Diego, CA USA; 3 Translate Bio, a Sanofi Company, Lexington, MA USA; 4 Sanofi Pasteur, Orlando, FL USA; 5 Pioneering Medicines, Cambridge, MA USA; 6 Seqirus Inc, Cambridge, MA USA; 7 Sanofi, Framingham, MA USA; 8 Sanofi Pasteur, Swiftwater, PA USA; 9 Texas Therapeutics Institute, The University of Texas Health Science Center at Houston, Houston, TX USA; 10 Sanofi Pasteur, Marcy l’Étoile, France

**Keywords:** Viral infection, Preclinical research

## Abstract

Recent approval of mRNA vaccines for emergency use against COVID-19 is likely to promote rapid development of mRNA-based vaccines targeting a wide range of infectious diseases. Compared to conventional approaches, this vaccine modality promises comparable potency while substantially accelerating the pace of development and deployment of vaccine doses. Already demonstrated successfully for single antigen vaccines such as for COVID-19, this technology could be optimized for complex multi-antigen vaccines. Herein, utilizing multiple influenza antigens, we demonstrated the suitability of the mRNA therapeutic (MRT) platform for such applications. Seasonal influenza vaccines have three or four hemagglutinin (HA) antigens of different viral subtypes. In addition, influenza neuraminidase (NA), a tetrameric membrane protein, is identified as an antigen that has been linked to protective immunity against severe viral disease. We detail the efforts in optimizing formulations of influenza candidates that use unmodified mRNA encoding full-length HA or full-length NA encapsulated in lipid nanoparticles (LNPs). HA and NA mRNA-LNP formulations, either as monovalent or as multivalent vaccines, induced strong functional antibody and cellular responses in non-human primates and such antigen-specific antibody responses were associated with protective efficacy against viral challenge in mice.

## Introduction

mRNA vaccines have reached a new milestone with clinical efficacy demonstrated for COVID-19 vaccines^1,2^ and dozens more vaccine candidates have entered the clinical stage of development^3–8^. In vitro transcribed mRNA packaged in LNPs can produce complex antigens in vivo, via engagement of endogenous ribosomal machinery, with proper post-translational modifications including assembly, glycosylation and trafficking resembling the native antigens. This vaccination modality conveys the advantages of live attenuated vaccines by inducing adaptive immune responses of both humoral and T cell-mediated immunity^9^. Unlike DNA vaccines, mRNA incurs minimal risk of host genome integration^10^. The unprecedented rapid development and approval of COVID-19 mRNA vaccines under Emergency Use Authorization, and the growing safety database in millions of human populations, support the exploration of the mRNA platform to address both global pandemic and seasonal viral pathogens.

Seasonal influenza virus A and B, with nearly 3–5 million annual cases and 0.29–0.65 million deaths worldwide, are global public health hazards^11^. Current seasonal influenza vaccines include two influenza A subtypes (H1N1 and H3N2) and two influenza B lineages (Victoria and Yamagata) as selected bi-annually by the World Health Organization. In addition, new zoonotic sources of influenza virus including those of avian and swine origins pose pandemic concerns highlighted by avian flu in southeast Asia in 2005, and global H1N1 swine flu in 2009^12^. Several subtypes of seasonal virus occur in nature with frequent mutation and reassortment resulting in antigen drift and shift. Mutations in the HA protein leads to the emergence of new viral variants, that may no longer be neutralized by antibodies to the seasonal HA protein in the vaccine, requiring seasonal updating of the vaccine to cover the most relevant circulating strains^13,14^.

A majority of currently marketed influenza vaccines are produced in eggs, a process that is not only vulnerable to disruptions in the egg supply, as reported in past avian influenza outbreaks^15^, but also results in egg-adaptation which may result in adaptive mutations in HA leading to diminished vaccine effectiveness^16^. A key challenge in the vaccine virus selection is that certain strains of influenza virus appear very late in the season after manufacturers have already started growing the vaccine virus^17^. An mRNA platform with reduced time to market not only allows time for more data collection prior to strain selection but would also eliminate the issues of egg dependence and host adaptation. Furthermore, an mRNA platform offers a generic manufacturing schema with the possibility to adapt to *in silico* designs that promise increased vaccine breadth and effectiveness^18^.

Although the mRNA technology has been used to study monovalent influenza HA antigens of seasonal or pandemic strains^19–23^, there is limited data, to our knowledge, on multivalent combinations of antigens. Here, we optimized the MRT platform for multivalent antigen combinations and demonstrate the utility in development of multivalent mRNA vaccines for seasonal or pandemic influenza. A panel of unmodified mRNAs including A/California/07/2009 influenza virus H1 HA (Cal09 HA), A/Singapore/INFIMH160019/2016 Influenza virus H3 HA (Sing16 HA), A/Singapore/INFIMH160019/2016 Influenza virus N2 NA (Sing16 NA), A/Michigan/45/2015 Influenza virus N1 NA (Mich15 NA), A/Perth/16/2009 Influenza virus N2 NA (Perth09 NA), and reporter antigens of Firefly luciferase (FF) and human erythropoietin (hEPO) were prepared. LNP formulations for HA and NA mRNA-LNP preparation were then tested for in vitro expression in vivo immunogenicity and potency in preclinical models.

## Results

### mRNA antigen preparation, characterization, and expression

mRNAs coding for the full-length codon-optimized HA and NA for the various influenza strains were synthesized enzymatically using unmodified ribonucleotides. All mRNA preparations had > 95% of 5′ Cap1 and showed a single homogenous peak on capillary electrophoresis (Supplementary Fig. 1a). mRNA-LNP formulations were prepared by mixing the various lipid components with mRNA under controlled conditions and at fixed ratios. All mRNA-LNPs exhibited >95% encapsulation with uniform hydrodynamic radius ranging from 95 to 105 nm and a poly dispersity index (PDI) of 0.060–0.136 (Supplementary Table 1). Cryo-electron microscopy (Cryo-TEM) of the Cal09 HA mRNA-LNP images showed uniform spherical particles with a multi-lamellar inner core structure. (Supplementary Fig. 1b). The lamellarity of the solid core structure analyzed further with Fourier Transform, indicated a 3.7 nm periodicity between layers. The uniform morphology of the particles seen in the micrographs are indicative of homogenous LNP preparations with proper assembly.

**Fig. 1:**
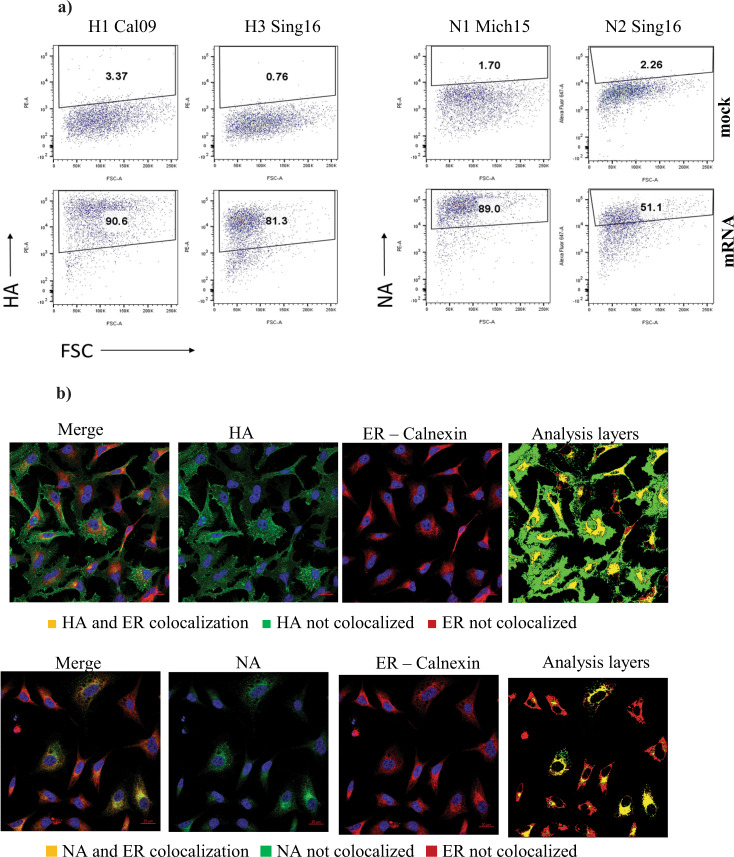
In vitro expression and localization of HA and NA in HskMC and HeLa cells transformed with mRNA. **a** Frequency of HA and NA expressing human skeletal muscle cells were evaluated by flow cytometry. HskMC cells were transfected with mRNA coding for Cal09 HA, Sing16 HA, Mich15 NA and Sing16 NA. Corresponding antigen expression was confirmed by intracellular staining with each antigen specific mouse or rabbit antibodies followed by fluorochrome conjugated goat anti-mouse IgG or goat anti-rabbit IgG secondary antibody. The numbers represent the frequency of antigen expression as determined by comparison with mock transfected HskMCs. For gating strategy see Supplementary Fig. 2. **b** Intracellular localization of expressed HA and NA in HeLa cells. HA and NA protein expression for a bivalent H3N2-mRNA LNP analyzed along with Calnexin an endoplasmic reticulum marker. Image panels show merged image of HA and ER (upper) or NA and ER (lower) antibody staining. Middle two images display separate HA and NA antibody staining (green) and ER marker Calnexin (red). Images in the analysis layer column display results of image analysis to measure colocalization of HA or NA with ER marker. Images contain image analysis mask layers to denote regions of HA or NA colocalization with ER (yellow), HA or NA not colocalized with the ER (green), and ER not containing any HA or NA expression (red).

To confirm antigen expression, HEK293FT cells were transfected with unencapsulated mRNA constructs of Cal09 HA, Sing16 HA, Sing16 NA, or Mich15 NA, and the expression of protein was confirmed by western blot. HA monomer of 75 kDa and NA monomer 60 kDa were identified under the reducing and denaturing condition (Supplementary Fig. 1c). Antigen expression was also confirmed with flow cytometry by transiently transfecting human skeletal muscle cells (HskMCs) with the unencapsulated mRNA constructs of Cal09 HA, Sing16 HA, Sing16 NA, or Mich15 NA, and stained with protein-specific antibodies for analysis. High levels of HA and NA expression from HskMCs were observed, confirming proper assembly and trafficking of native form HA and NA upon expression in muscle cells (Fig. 1a, Supplementary Fig. 2). To study the subcellular localization of expressed HA and NA proteins, HeLa cells were transfected with bivalent H3N2 LNP and proteins were visualized by immunostaining and confocal microscopy. While the NA signal indicated strong colocalization in endoplasmic reticulum (ER) (~90%), HA was found to colocalize moderately with ER when permeabilized cells were stained with antibodies for corresponding proteins and Calnexin, an ER marker **(**Fig. 1b). This is consistent with the understanding that nascent NA and HA proteins are translocated to the ER for assembly^24^.

The efficiency of delivery of mRNA by LNPs and selection of optimal formulation parameters was evaluated using reporter mRNA expression^25^. A single dose of either 0.05, 0.1, 1, or 5 μg of unmodified FF-LNP formulations was administered intramuscularly (IM) in mice. Luciferase activity, measured by average bioluminescence, indicated sustained expression from the mRNA construct which peaked at 6 h post injection and was detectable beyond 72 h at all doses (Supplementary Fig. 3a, b). The high-level mRNA-mediated protein expression was further verified with hEPO at a single 0.1 μg dose in mice and 10 μg in non-human primate (NHP). The study was intended to compare LNP, using standard LNP Dlin-MC3-DMA^26^ formulation as a control. Serum hEPO quantified by ELISA demonstrated maximum expression at 6 h with ~12-fold higher erythropoietin expressed with hEPO-LNP compared to hEPO-MC3 (Supplementary Fig. 3c). Both hEPO-LNP and hEPO-MC3 showed similar expression kinetics in NHPs, detectable from 6 h to 72 h (Supplementary Fig. 3d). The results confirmed the efficient delivery of mRNA by the MRT LNP formulation in both in vitro and in vivo models.

### Immunogenicity of HA (H1, H3) and NA (N1, N2) mRNA-LNP in mice

**Fig. 2:**
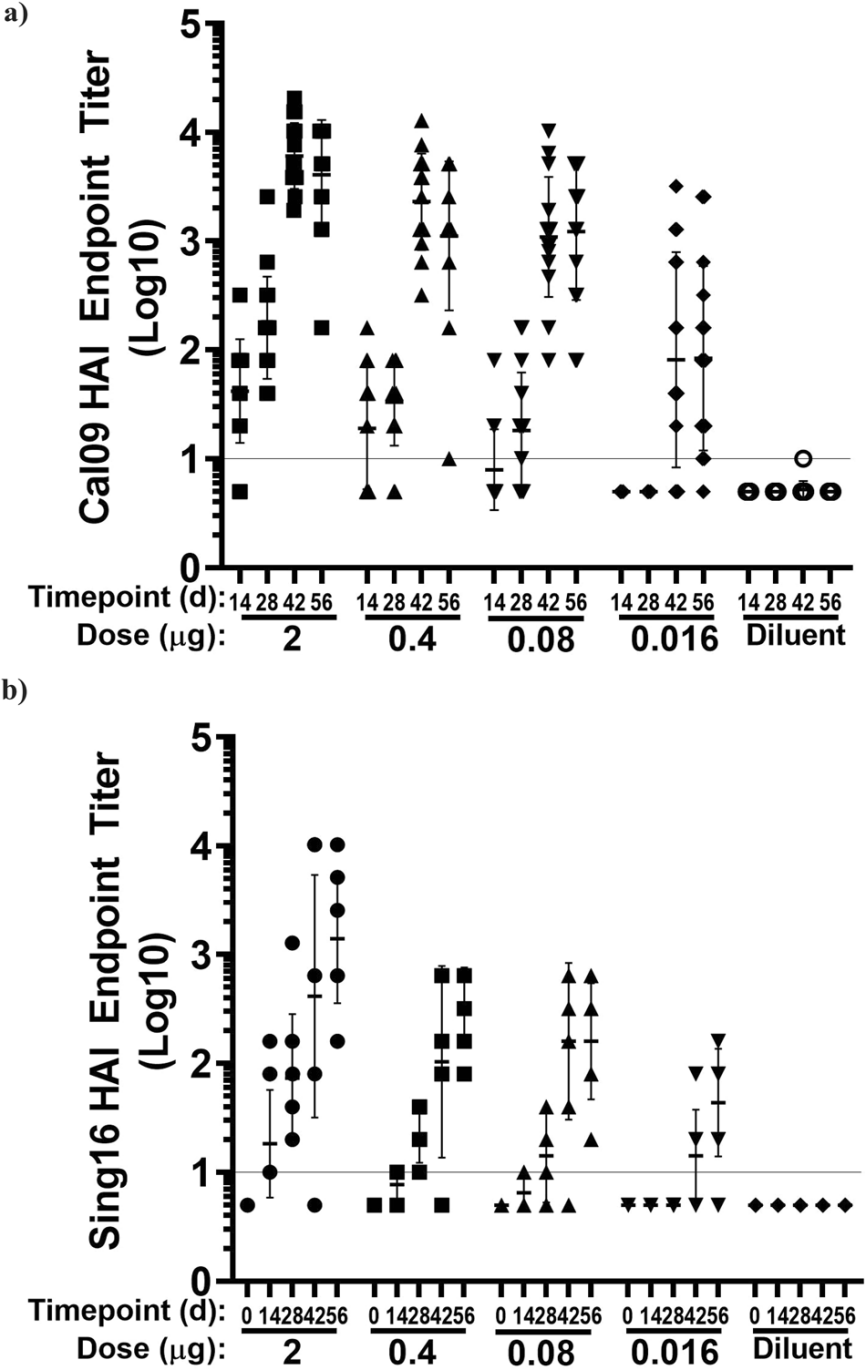
Serological Evaluation of HA mRNA-LNP vaccine in mice. BALB/c mice (*n* = 8 per group) were immunized twice IM, 4 weeks apart with 2, 0.4, 0.08 and 0.016 μg of either Cal09 HA mRNA-LNP or Sing16 HA mRNA-LNP. Log10 HAI titers recorded against (**a**) A/California/07/2009 H1N1 influenza virus (**b**) A/Singapore/INFIMH160019/2016 H3N2 influenza virus shown. Each dot represents an individual animal, and the line represents the geometric mean for the group. Lower horizontal line in each panel represents the lower limit of assay read out. See Supplementary Fig. 4 for ELISA titers.

Natural history and vaccine studies have shown that antibodies to influenza HA and NA have antiviral function and both antigens are considered important for effective influenza vaccines^14^. Unmodified Cal09 HA-LNP and Sing16 HA-LNP mRNA vaccines were evaluated in BALB/c mice (*n* = 8) in a two-dose regimen at 2, 0.4, 0.08, or 0.016 μg mRNA-LNP administered at 4-week apart schedule. Recombinant HA (rHA) antigens of the same strain were used to evaluate the total IgG responses in ELISAs. HA-specific antibodies were detected in all groups after a single dose, but the titers peaked at day 42 after the second dose (Supplementary Fig. 4). To measure functional antibodies, hemagglutination inhibition (HAI) response was evaluated against the homologous strains, A/California/07/2009 H1N1 and A/Singapore/ INFIMH160019/2016 H3N2. Although the HAI titers after a first dose could be observed for the 2 μg dose of Cal09-LNP and Sing16-LNP treatment groups with Geometric Mean Titers (GMTs) of 160 and 70 at day 28 respectively, a more profound increase in HAI titers was observed after the second dose. At day 42, GMT titers were 80 and 2,200 for the 0.016 μg and 0.4 μg groups, respectively, in the Cal09 -HA-LNP and 14 and 100 for the 0.016 μg and 0.4 μg groups, respectively, in the Sing 16 HA-LNP groups (Fig. 2a, b).

**Fig. 3:**
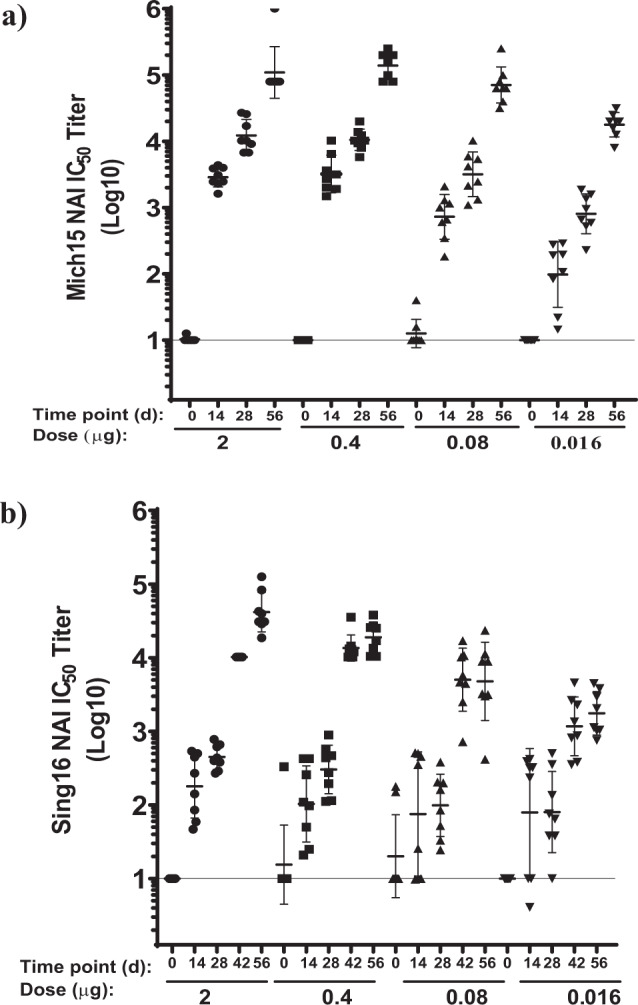
Serological Evaluation of NA mRNA-LNP vaccine in mice. BALB/c mice (*n* = 8 per group) were immunized twice IM 4 weeks apart with 2, 0.4, 0.08, and 0.016 μg of either Mich15 NA mRNA-LNP or Sing16 NA mRNA-LNP. Log10 NAI (ELLA) titers recorded for sera against (**a**) A/Michigan/45/2015 (N1): A/Mallard/Sweden/2002 (H6) chimeric influenza virus, **b** A/Singapore/INFIMH160019/2016 (N2): A/Mallard/Sweden/2002 (H6) chimeric virus are depicted. Each dot represents an individual animal, and the line represents the geometric mean for the group. Lower horizontal line in each panel represents the lower limit of assay read out. See Supplementary Fig. 5 for ELISA titers.

Similarly, for testing anti-NA responses, mice were immunized with 2, 0.4, 0.08, or 0.016 μg of Sing16 NA-LNP or Mich15 NA-LNP. ELISA with recombinant NA (rNA) antigens were conducted to assess the total IgG responses induced by either Mich15 NA-LNP or Sing16 NA-LNP formulations. Animals developed high antibody binding responses after a single dose, with a marked increase in NA binding antibodies post second dose at day 42 (Supplementary Fig. 5). Enzyme-linked lectin assay (ELLA) was used as a surrogate for functional antibody titers for Neuraminidase inhibition (NAI) activity against H6N1 or H6N2 chimeric viruses. Although two doses of the vaccine substantially increased the functional antibody response as compared to a single dose, encouraging NAI titers with GMTs 800 and GMT 60 were recorded at day 28 after a single dose even with low dose of 0.016 μg of Mich15 NA-LNP and Sing16 NA-LNP, respectively. At day 42, the GMT titers for 0.4 μg and 0.016 μg, were 10,200 and 900 respectively in the Sing16 NA-LNP group indicating a dose-dependent response with titers reaching above upper limit of detection (ULOQ = 10,240) in the case of Mich15 NA-LNP (Fig. 3a, b).

### Protection from viral challenge in mice

**Fig. 4:**
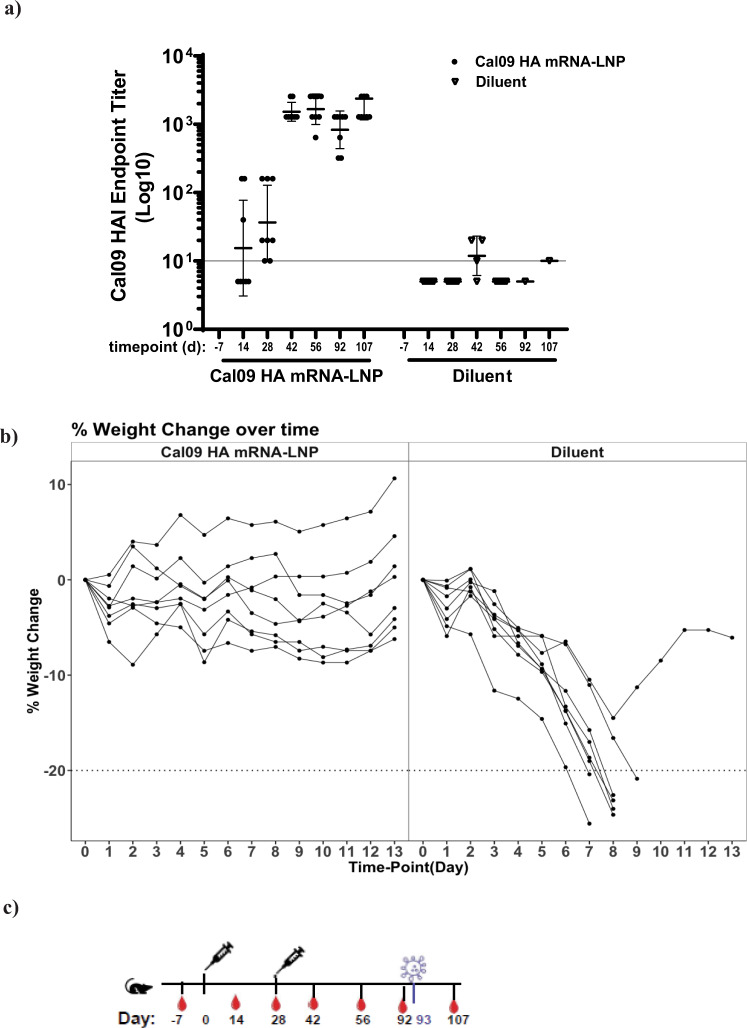
Protective efficacy of Cal09 HA mRNA-LNP vaccine in mice after lethal A/Belgium/2009 H1N1 virus challenge. Mice (*n* = 8) received two IM doses of Cal09 HA mRNA-LNP (0.4 μg each) on day 0 and day 28. Control animals received two IM doses of diluent on day 0 and day 28. **a** HAI titers are reported as Log10 for serum samples taken at study days -7, 14, 28, 42, 56, 92, 107 are reported. Each dot represents an individual animal, and the line represents the geometric mean for the group. Lower horizontal line represents the lower limit of assay read out. **b** Daily weights after intranasal challenge with 4LD50 of A/Belgium/2009 H1N1 strain on day 93 are reported. Weights are presented as the percentage of weight lost from the day of challenge. Individual lines represent each animal. **c** Immunization and challenge schedules are provided.

To test the efficacy of the mRNA vaccines in a mouse influenza virus challenge model, we inoculated BALB/c mice with 0.4 μg of Cal09 HA-LNP IM at week 0 and 4, along with a negative control group with two doses of LNP diluent buffer. HAI titers for vaccine group serum samples collected at study days 0, 14, 28, 42, 56, 92, and 107 demonstrated robust immune response with GMT of 1,660 and 830 at day 56 and day 92, respectively (Fig. 4a). At day 93, all mice were challenged intranasally with A/Belgium/145-MA/2009 H1N1 virus, homologous to A/California/07/2009, at four times the dose which can cause 50% lethal outcome (4xLD_50_). All mice in the vaccine group survived the challenge with no mortality, and some mild morbidity marked by transient weight loss of less than 5% (Fig. 4b). However, mice in the diluent control group suffered significant and rapid weight loss which led to high mortality rate (90%) by day 9. These results demonstrated high efficacy of HA-based MRT formulations in a lethal mouse influenza challenge model.

**Fig. 5:**
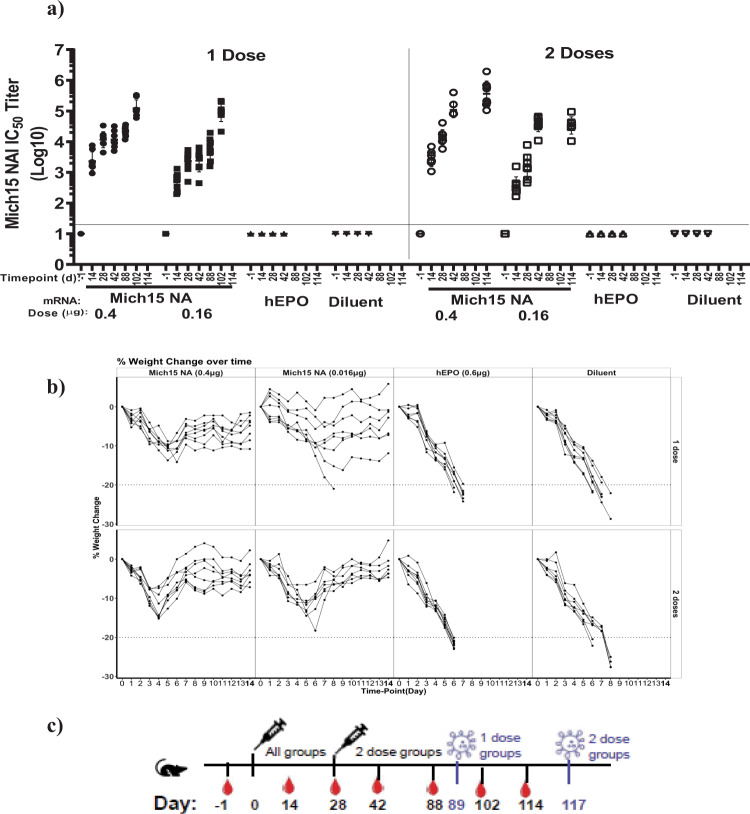
Protective efficacy single dose of unmodified Mich15 NA mRNA-LNP in mice after lethal A/Belgium/2009 H1N1 virus challenge. Mice (*n* = 16) were injected by the IM route with 0.4 μg or 0.016 μg of A/Michigan/45/2015 NA mRNA-LNP. Half of the mice only received one injection (1 dose) on study day 0, while the other half (2 doses) received two injections given at study day 0 and day 28. Control animals received two IM doses of diluent or hEPO mRNA-LNP (0.6 μg) on day 0 and day 28. **a** NAI titers are reported as Log10 for serum samples taken at study days -1, 14, 28, 42, 88, 102 for one dose group; -1, 14, 28, 42, 114 for 2 dose group and -1, 14, 28, 42 for control group. Each dot represents an individual animal, and the line represents the geometric mean for the group. Lower horizontal line represents the lower limit of assay read out, **b** daily weight change after intranasal challenge on day 89 for single dose group and day 117 (89 days after second dose) for two dose group with 4LD_50_ of Belgium09 H1N1 are reported. Weights are presented as the percentage of weight lost from the day of challenge. Individual lines represent each animal. **c** Immunization and challenge schedules are provided.

To assess the protective efficacy of NA-based MRT vaccines, we conducted an analogous challenge experiment in BALB/c mice. Since the Mich15 NA-LNP vaccine elicited robust NAI titers after a single immunization in naive mice, one and two dosing regimens with administrations of 0.4 or 0.016 μg of Mich15 NA-LNPs over a 4-week interval were evaluated. The control groups were vaccinated at the same regimens, receiving either 0.6 μg hEPO-LNP or diluent buffer. Robust NAI titers were observed after a single administration with GMTs of 14,000 NAI for 0.4 μg and 1,800 NAI for 0.016 μg of Mich15 NA-LNP recorded at day 28 (Fig. 5a). After the second immunization at day 42, NAI titers rose to 108,000 NAI for 0.4 μg and 37,000 NAI for 0.016 μg groups. After about 9-12 weeks post-vaccination regimens, all groups were challenged with 4xLD_50_ of A/Belgium/145-MA/2009 H1N1 virus. Individual weight changes from baseline over time by treatment groups are graphed in Fig. 5b. All mice in the two control groups suffered significant morbidity, and all animals had to be euthanized due to >20% weight loss by day 8 post-infection. Notably, all animals except one in the vaccine groups survived the challenge in the single dose 0.016 μg group, indicating high protective efficacy against death even after a single dose of as low as 0.016 μg of Mich15 NA-LNP. The higher dose (0.4 µg) demonstrated overall higher protection, however, in contrast to HA-immunization, NA vaccination was not sufficient to protect against weight loss as vaccinated animals demonstrated median weight loss of 10 % of initial body weight. This is rather consistent with observations reported for other NA vaccines that while HA specific antibodies prevent infection by blocking the HA-mediated attachment to the cell surface the NA-specific antibodies limit vial load by blocking the exit of virions leading to reduction of clinical symptoms but not necessarily preventing infection^27^. Body weight recoveries were observed for vaccinated groups resulting in an average final weight change of 2.7% at the low dose and 4.8% weight gain for the higher dose, as compared to baseline. Overall, the results demonstrated that a single low-dose MRT NA-LNP vaccination can elicit functional antibodies measurable for blocking influenza NA activity and sufficient to confer protection against lethal challenge in mice.

### Immunogenicity of HA (H3) mRNA-LNP in NHP

**Fig. 6:**
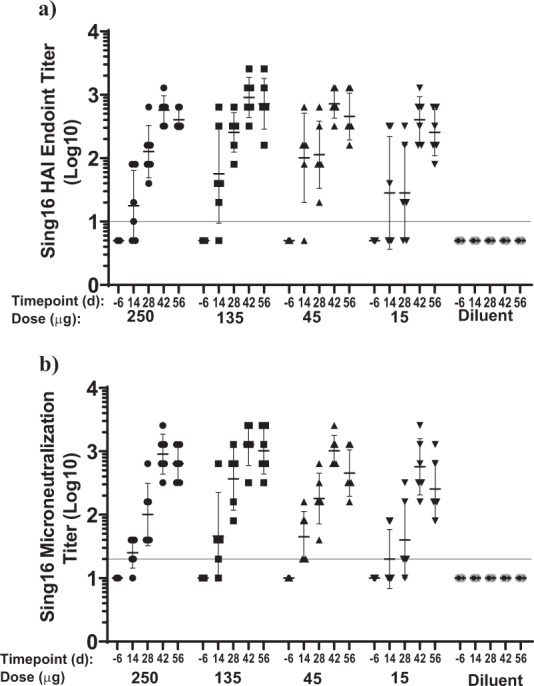
Serological Evaluation of HA Sing16 HA mRNA-LNP vaccine in NHP. Cynomolgus macaques (*n* = 6 per group) were injected twice, 4 weeks apart by IM route, with 15, 45, 135, or 250 μg of Sing16 HA mRNA-LNP. Serum samples were collected at days -6, 14, 28, 42, and 56. (**a**) Log 10 HAI titers against A/Singapore/INFIMH160019/2016 virus and (**b**) Log 10 micro neutralization (MN) titers against A/Singapore/INFIMH160019/2016 virus are shown. Each dot represents an individual animal, and the line represents the geometric mean for the group. Lower horizontal line represents the lower limit of assay read out. See Supplementary Fig. 6 for ELISA titers against recombinant Sing16 HA protein and Supplementary Table 2 for statistical analysis.

To evaluate immunogenicity of the mRNA-LNP in NHP, a dose range study covering 15, 45, 135, and 250 μg of Sing16 HA-LNP was performed in NHPs. After the first immunization, all vaccinated NHPs developed antibodies reactive to recombinant HA protein as noted in ELISA (Supplementary Fig. 6, Supplementary Table 2). Further boosting of titers was observed post second dose in all groups except the 250 μg group. From day 14 to day 42 a statistically significant dose effect between the four doses was observed, however, this was not the case for day 56. A pair wise dose by dose comparison interestingly indicated that the 15 μg dose induced only 1.8-fold lower ELISA titers than the 135 μg dose level (95% CI 1.0, 3.6), suggesting a dose saturation close to 15 μg level. Analysis of the HAI responder groups showed a significant boost effect between day 28 and day 42 in all dose groups but no statistically significant differences between the various dose levels on day 14, 42, and 56. Robust HAI antibodies were induced in all dose groups on day 42 and GMTs recorded were 400 for 15 μg, 700 for 45 μg, 900 for 135 μg and 570 for 250 μg. At day 42, the fold increase in GMT titers with 95% CI was 2.2-fold (1.0; 5.0) between the 135 μg and 15 μg and was 1.3-fold (0.6; 2.8) between the 135 μg and 45 μg treatment groups indicating that despite the observed trend towards higher titers with increasing dose, the difference between groups was minimal (Fig. 6a, Supplementary Table 2). The neutralization potency assessed by microneutralization (MN) assay indicated a significant boost effect between day 28 and day 42 (Fig. 6b, Supplementary Table 2) showing a better trend for dose response with GMTs on day 42 of 570 for 15 µg, 1,016 for 45 µg, 1,280 for 135 µg and 905 for 250 µg.

**Fig. 7:**
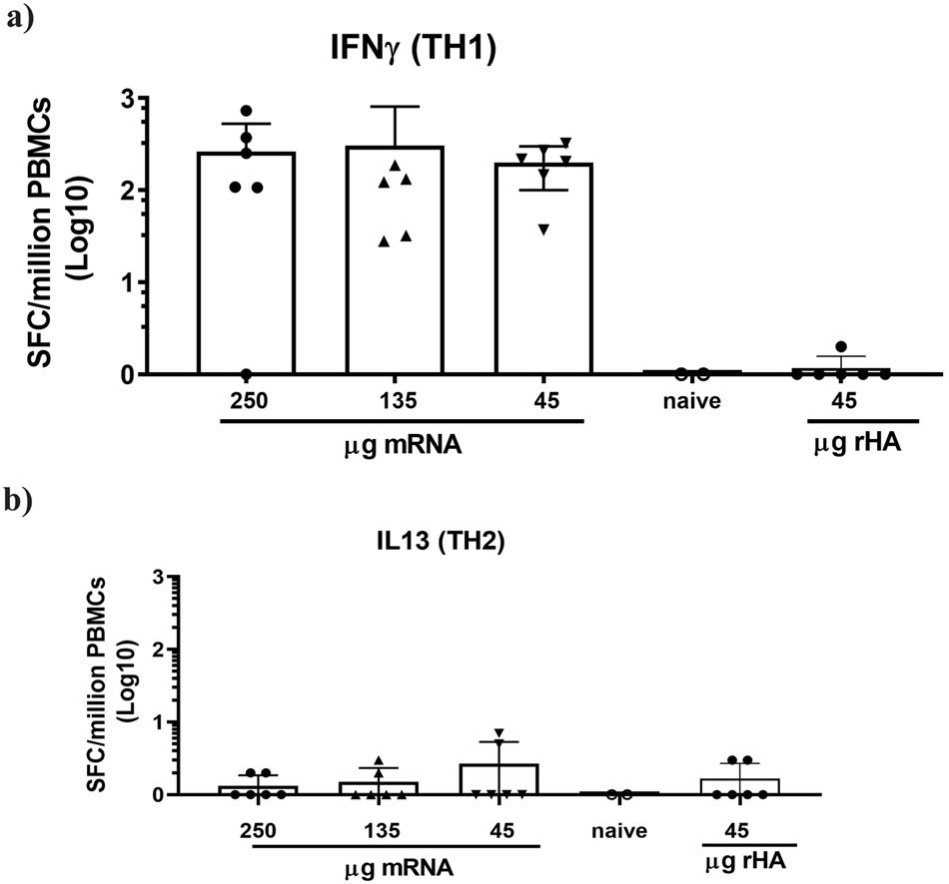
T cell responses in NHP vaccinated with Sing16 HA mRNA-LNP vaccine. Cynomolgus macaques (*n* = 6 per group) were injected twice, 4 weeks apart by IM route, with 45, 135, or 250 μg of Sing16 HA mRNA-LNP. T cells were determined by ELISPOT on day 42 in PBMC stimulated in vitro with peptide pools to represent the entire HA open reading frame. The responses of PBMC secreting IFNɣ (**a**) or IL-13 (**b**) calculated as spots forming cells (SFC) per million PBMC are shown. Each symbol represents an individual sample, and the bar represents the mean with standard deviation for the group.

Since T cells have been shown effective in reducing viral load and limiting disease severity in animal models,^28,29^ we evaluated recall T cells in the NHPs vaccinated with 45, 135, 250 μg of Sing16 HA-LNP or with 45 μg of rHA protein. Primary Blood Mononuclear Cells (PBMCs) collected at day 42 were evaluated in IFN-γ (Th1 cytokine) and IL-13 (Th2 cytokine) ELISPOT assay with recall stimulation with pooled overlapping peptides spanning the entire sequence of the Sing16 HA protein. All vaccinated animals except one in 250 μg group developed IFN-γ secreting cells, ranging from 28 to 1,328 spot-forming cells (SFC) per million PBMCs (Fig. 7a). Notably, a dose–response was not observed, and the lower and higher dose level groups of animals showed comparable frequencies of IFN-γ secreting cells. In contrast, all animals in the control group immunized with the recombinant Sing16 HA protein demonstrated the absence of IFN-γ producing cells. The presence of IL-13 cytokine secreting cells were either not detected or very low in all the groups tested (Fig. 7b). The data suggest that Sing16 HA-LNP induced strong Th1-biased cellular responses in NHPs, comparable to that seen with MRT5500^30^, the SARS-COV-2 vaccine, currently under development.

**Fig. 8:**
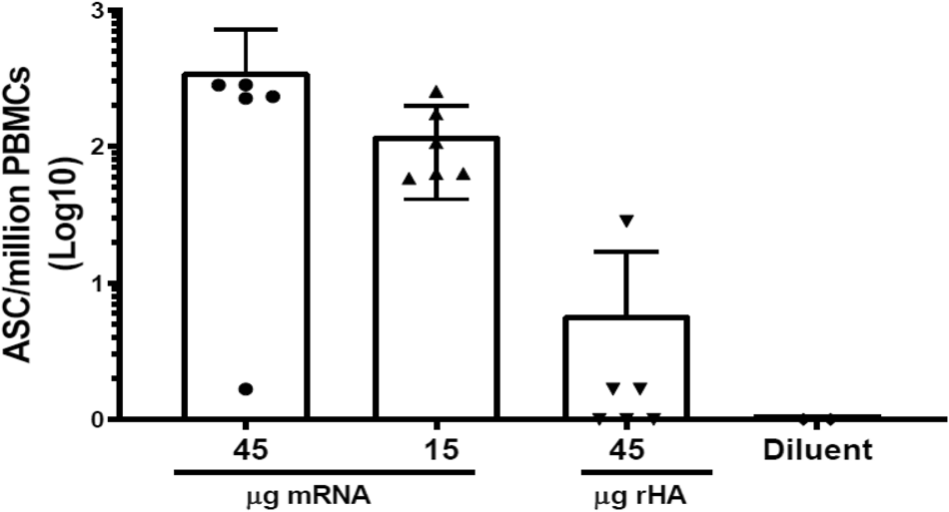
Secretion of A/Singapore/16/H3-specific IgG by memory B cells on day 180 in NHP vaccinated with Sing16 HA mRNA-LNP vaccine. Cynomolgus macaques (*n* = 6 per group) were injected twice, 4 weeks apart by IM route, with 15 or 45 μg of Sing16 HA mRNA-LNP. The Human IgG Single-Color memory B cell ELISPOT kit (CAT# NC1911372, CTL) was used to measure A/Singapore/16/H3-specific and total IgG+ antibody-secreting cells (ASCs). Differentiation of MBCs into ASCs was performed in PBMC collected at day 180 by using a stimulation cocktail provided by the kit. The number of IgG+ and number of A/Singapore/16/H3-specific ASCs was calculated per million of PBMCs for each animal and the frequency of antigen-specific ASCs is shown. Each symbol represents an individual sample, and the bar represents the mean with standard deviation for the group. The percentage of antigen-specific ASCs to the total IgG+ ASCs is shown in Supplementary Table 3.

To investigate frequency of memory B cells (MBCs) in NHPs after immunization with Sing16 HA-LNP, an ELISPOT assay was developed to quantify antigen-specific MBCs as a readout of humoral immune memory. On day 180, PBMCs were collected from the NHPs immunized with 45 μg or 15 μg of the Sing16 HA mRNA-LNP formulations or with a recombinant HA as a comparator at a 45 μg dose. A 4-day polyclonal stimulation of PBMCs that is optimized to drive memory B cells to antibody secreting cells (ASC), and the stimulated PBMCs were plated in an antigen-specific ELISPOT where the frequency of antigen-specific ASCs could be determined. Antigen-specific memory B cells were then quantified as a percentage of total IgG+ memory B cells. Antigen-specific memory B cells were detected in all animals and their frequency ranged from 1 to 5% for the 45 ug dose group and 0.3 to 1.5% for the 15 μg dose group (Supplementary Table 3). In the rHA immunized animals, the memory B cell responses appeared to be markedly lower as antigen-specific memory B cells were undetectable in five out of six animals (Fig. 8) indicating that Sing16 HA-LNP like other mRNA vaccines elicits antigen specific memory B cells responses that promise to prolong immunity^31^.

### Multivalent influenza virus antigens

**Fig. 9:**
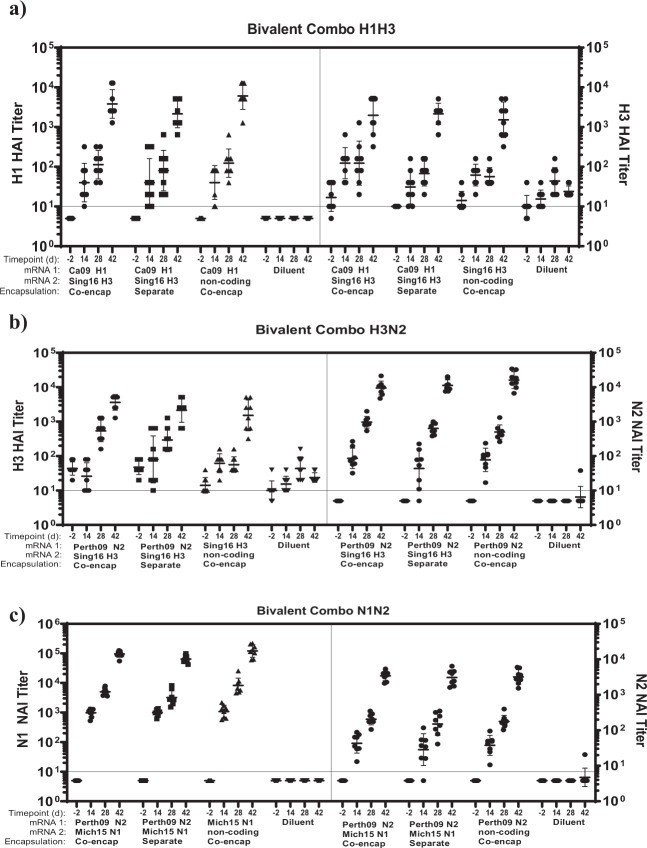
Delivery of bivalent combinations of influenza vaccine in mice. BALB/c mice (*n* = 8 per group) were immunized twice IM, 4 weeks apart with a total 0.4 μg of bivalent combinations co-encapsulated mRNA transcripts (1:1 wt/wt, half volume per each leg) or 0.2 μg each monovalent which was separately formulated and immunized different legs. H1H3 combo constituting Cal09 HA mRNA-LNP, Sing16 HA mRNA-LNP; H3N2 combo of Sing16 HA mRNA-LNP and Perth09 mRNA-LNP and N1N2 combo of Mich15 NA mRNA-LNP and Perth09 NA mRNA-LNP were tested in sera collected a day -2, 14, 28, 42, against corresponding virus. Control treatments included diluent or bivalent co-encapsulated formulations of corresponding HA or NA mRNA with non-coding mRNA. **a** HAI titers recorded against A/California/07/2009 H1N1 influenza virus and A/Singapore/INFIMH160019/2016 H3N2; **b** HAI and NAI titers recorded against A/Singapore/INFIMH160019/2016 H3N2 and A/Mallard/Sweden/2002 (H6) chimeric influenza virus and H6N2 A/Perth/09 virus (N2) virus, respectively (**c**). NAI titers recorded against A/Mallard/Sweden/2002 (H6) chimeric influenza viruses H6N1 A/Michigan/45/2015 (N1) and H6N2 A/Perth/09 virus (N2) virus are shown for each combination. Each dot represents an individual animal, and the line represents the geometric mean for the group. Lower horizontal line represents the lower limit of assay read out. See Supplementary Table 4 for design of study and Supplementary Table 5 for statistical analysis.

One of the advantages of the mRNA-LNP platform is the flexibility of LNP encapsulation for multiple mRNA antigen constructs. However, this potential needs to be tested to address the concern of antigenic interference. To explore combinations of influenza antigens, co-encapsulated HA and NA bivalent mRNA LNPs containing 0.2 μg each of mRNA in a H3H1, H3N2, or N1N2 combination were tested alongside monovalent LNP containing 0.2 μg of corresponding each antigen (Supplementary Table 4). These formulations were administered in mice to determine any antigenic interference on immunogenicity by comparing the functional titers of the individual antigen in bivalent vs. monovalent formulations (Fig. 9a–c, Supplementary Table 5). In the H1H3 combo, no statistical difference (*p* = 0.2584) for H1 HAI titers was seen between the co-encapsulated and separately administered formulations irrespective of the time points and no significant difference (*p* = 0.8389) at day 42 was seen for H3 titers. In the case of H3N2 combo, the NA component of the vaccine elicited high neutralizing antibodies in combination with the HA component demonstrating lack of HA dominance. Between the co-encapsulated and separately administered vaccines no statistically significant difference (*p* = 0.2960) irrespective of the time points was seen for H3 HAI titers, and no significant difference (*p* = 0.0904) at day 42 was seen for N2 NAI titers. Likewise, the N1N2 combo was not statistically different (*p* = 0.3899) for N2 NAI titers. N1 NAI titers at day 42 for co-encapsulated vaccine were higher (*p* = 0.0291) than the separately administered vaccines. In summary, bivalent combinations of N2N1, H3H1, or H3N2 generated antibody titers equivalent to individual separately formulated LNPs.

**Fig. 10:**
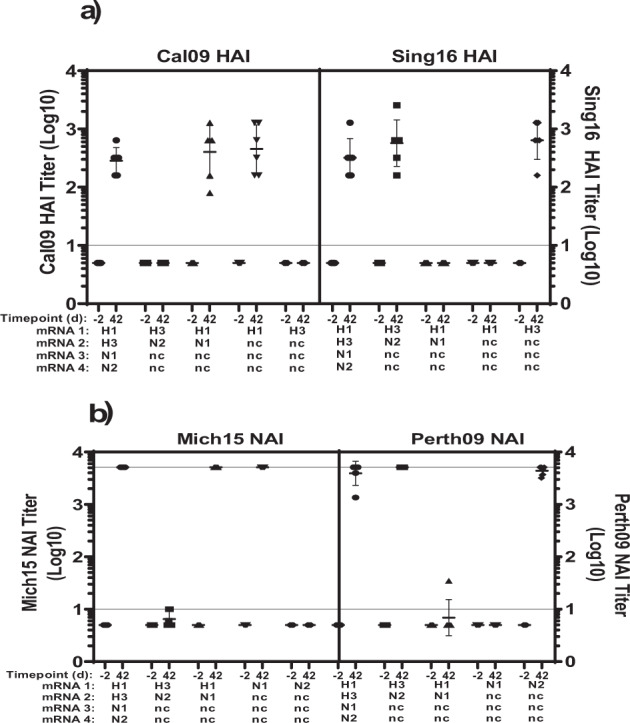
Delivery of quadrivalent combinations of influenza vaccines in NHP. Cynomolgus macaques (*n* = 6 per group) were immunized twice IM, 4 weeks apart with a total 10 μg of quadrivalent combinations of co-encapsulated mRNA transcripts (1:1:1:1 wt/wt). H1N1H3N2 combo consisting of Cal09 HA mRNA, Sing16 HA mRNA, Mich15 NA mRNA, and Perth09 NA mRNA. H1H3 combo constituting Cal09 HA mRNA, Sing16 HA mRNA and 2x non-coding mRNA (ncmRNA); H3N2 combo of Sing16 HA mRNA and Perth09 NA mRNA and 2x non-coding mRNA. N1N2 combo of Mich15 NA mRNA, Perth09 NA mRNA-LNP, and 2x non-coding mRNA served as bivalent controls. H1 consisting of Cal09 HA mRNA and 3x non-coding mRNA; H3 consisting of Sing16 HA mRNA and 3x non-coding mRNA; N1 consisting of Mich15 NA mRNA and 3x non-coding mRNA and N2 consisting of Perth09 NA mRNA and 3x non-coding mRNA served as monovalent controls. Inhibitory titers were tested in sera collected a day -2 and 42, against corresponding virus - (**a**) HAI titers recorded against A/California/07/2009 H1N1 Influenza virus and A/Singapore/INFIMH160019/2016 H3N2; (**b**) NAI titers recorded against A/Michigan/45/2015 (N1): A/Mallard/Sweden/2002 (H6) chimeric influenza virus and H6N2 A/Perth/09 virus F1919D (N2): A/Mallard/Sweden/2002 (H6) chimeric influenza virus is shown. are shown for each combination. Each dot represents an individual animal, and the line represents the geometric mean for the group. Lower horizontal line represents the lower limit of assay read out. See Supplementary Table 6 for design of study and Supplementary Table 7 for statistical analysis.

We further explored quadrivalent formulations of co-encapsulated H1, N1, H3, and N2 mRNA. These formulations were tested in NHPs with a total of 10 μg mRNA composed of 2.5 μg of each influenza antigen mRNA and or filling with a corresponding amount of noncoding mRNA (nc mRNA) resulting in quadrivalent (H1N1H3N2), bivalent (H1N1 or H3N2), or monovalent (H1, H3, N1, or N2) LNPs (Supplementary Table 6). HAI titers to H1 or H3, or NAI titers to N1 or N2 were compared between the monovalent formulations vs. bivalent or quadrivalent formulations (Fig. 10, Supplementary Table 7). On day 42, the HAI titers to H1 of the quadrivalent group were comparable when analyzed with that of the H1 monovalent group (*p* = 0.9054, t-test, unpaired, two-tailed) or H1N1 bivalent group (*p* = 0.8002). Similarly, the H3 HAI titers of the quadrivalent group was comparable when analyzed with that of the H3 monovalent group (*p* = 0.2504) or H3N2 bivalent group (*p* = 0.5894). The NAI titers to N1 were almost identical in groups of animals vaccinated with N1 monovalent mRNA or H1N1 bivalent mRNA or the quadrivalent H1N1H3N2 mRNA formulations. Likewise, there was no difference in N2 NAI titers between the N2 monovalent mRNA (*p* = 0.8485) or H3N2 bivalent mRNA (0.4545) with the quadrivalent H1N1H3N2 mRNA formulations. Overall, these findings indicate that co-encapsulated and combined multivalent vaccines of HA/NA mRNA-LNPs could efficiently deliver all four antigens without any sign of immunological interference and all antigens were as immunogenic as in the formulation when these antigens were delivered singularly.

## Discussion

Several advancements in the design of mRNA have led to improvements in its translatability, stability, and immunomodulatory activities^32–34^. To express antigen efficiently, mRNA requires (i) careful selection of 5′ and 3′ UTRs responsible for recruiting RNA binding proteins; (ii) codon optimization algorithms to include synonymous and more frequent codons to replace rare codons for protein sequences; and (iii) modification of the 5′ cap to reduce the innate immune responses. The immunomodulatory profile of mRNA is influenced by (i) the purity of in vitro transcribed mRNA and particularly the content of dsRNA^35^ and/or (ii) introduction of modified nucleosides in the mRNA^36,37^. Contrary to the belief that innate immune sensing of unmodified mRNA could lead to inhibition of antigen expression and negatively affect the immune responses, we observed sustained production of protein comparable to modified RNA^21^ emphasizing the importance of purity and rational designs of RNA structure/sequence, in determining potency and performance of the vaccine^38^. With respect to LNP delivery systems, optimized molar ratios of the lipid components composed of (i) an ionizable lipid with cationic charge facilitating encapsulation of the mRNA and driving the dynamics of endosomal release; (ii) a zwitterionic lipid that aids membrane fusion; (iii) cholesterol as a stabilizer; and (iv) a PEG lipid aiding in protection and stability of the LNP were used to ensure enhanced cellular uptake and endosome escape^39,40^. Cryo-TEM images of the Cal09 HA-LNP showed uniform spherical particle structure, indicating that the LNP formulation is homogenous. The findings are consistent with morphology of mRNA-LNPs that have been reported previously^41^. The MRT platform provides an advanced technology that incorporates the elements of design for both the mRNA component and the delivery system making it suitable to develop vaccine candidates against a broad range of disease targets.

mRNA vaccines have been shown to induce strong and potent humoral and T cell responses against several infectious disease targets including SARS-COV-2^42,43^ cytomegalovirus^44^, zika virus^45^, respiratory syncytial virus^46^, human metapneumovirus^47^ as well as influenza virus^19^. The current standard of care for a seasonal influenza vaccine is a quadrivalent vaccine containing H3, H1, and B influenza strain antigens as selected by the WHO. We evaluated two model antigens: HA, the surface glycoprotein and the main target in current vaccines driving induction of neutralizing antibodies; and NA, a less abundant viral surface glycoprotein, antibodies to which are known to inhibit viral replication. We demonstrated here that HA and NA mRNA-LNPs, can generate robust and protective immune responses in mice. Several studies have previously reported anti-HA responses to mRNA vaccines in mice^48,49^. In our study, a single 2 μg dose of Cal09 HA-LNP resulted in GMT of 160 in mice, consistent with observations that a 3 μg dose of FPLC-purified full length Cal09 HA-encoding mRNA encapsulated in LNP resulted in >120 HAI titers and offered protection upon challenge^19^. Although we tested protection mediated by HA response after 2 immunizations of a Cal09 HA-LNP, an important outcome of our study is the finding that a single 16 nanogram dose of a Mich15 NA-LNP could provide a robust functional antibody response and protection from lethal challenge. Similar observations of protective low doses of NA mRNA in mice have been reported in a recent study^50^.

NA functions as an enzymatic protein which cleaves terminal sialic acid from glycans and helps the virus to penetrate the mucosal fluid to reach its target cells and also permits newly made budding viruses to release from the cell to then infect nearby cells, thus playing a crucial role in viral lifecycle^51^. It has been long proposed as a target for development of influenza vaccines^52^. Although current standard dose egg-based inactivated influenza virus vaccines contain low amounts of NA as a result of the production process, it is not quantified and thus a suboptimal dose to induce a robust immune response^53^. Thus far, there has been limited success to obtain GMP-grade purified or recombinant NA for demonstration of clinical efficacy and the mRNA platform offers an opportunity to provide an improved influenza vaccine formulation that expresses not only an HA protein but also a properly folded NA protein for induction of a robust neutralizing antibody response. Furthermore, mRNA-derived NA overcomes the immunodominance of HA often reported in combined recombinant HA, NA vaccination wherein lower immune responses against NA have been reported^54^.

Dose ranging studies in NHPs confirmed the potency of the Sing16 HA-LNP in eliciting neutralizing antibodies. We observed robust HAI titers, in NHPs, following single IM dose of 15 to 45 μg of Sing16 HA-LNP compared to 200 or 400 μg dose vaccination of H10 or H7 mRNA-MC3^21^. A minimal difference in dose responses in NHPs could be indicative of plausible dose sparing effect of MRT formulations. As all the animals assigned in the study were naive for influenza before immunization, we anticipate that the presence of cross-reactive HA-specific memory B cells and T cells in a clinical setting, with pre-existing HA immunity, will likely result in a stronger boosting response. It is encouraging to see the remarkably higher antigen-specific memory B cells circulating in NHPs 6 months after immunization with Sing16 HA-LNP compared to the subunit vaccine. Ongoing clinical studies with these LNPs will determine if such responses can be induced in humans as well. Spike protein-specific memory B cell responses in SARS-CoV-2 naive individuals following SARS-COV-2 mRNA vaccination^55^ and HA-specific memory B cell responses following mRNA influenza vaccination^56^ have been reported suggesting that mRNA vaccine platforms may have a better ability to induce long lasting B cell memory than a recombinant protein, potentially due to proper presentation of relevant neutralizing epitopes from de novo expressed antigens by mRNA. In addition, Sing16 HA-LNP vaccine induced strong Th1-biased responses in NHPs with antigen specific interferon-γ produced by large portion of HA-specific T cells consistent with the observations with other mRNA vaccines^42^. As cell-mediated immunity plays an important role in protection against influenza illnesses, hetero-subtypic immunity and in the establishment of memory it would be interesting to additionally assess the role of T cells in mediating broadly protective immunity, amidst reports of heterologous protection observed in recent Influenza mRNA vaccine studies^19,50^ in the absence of HAI titers.

With monovalent mRNA vaccines demonstrating high potency and protection in challenge models, we sought to further test this technology to include multivalent approaches. To this end, we formulated bivalent LNPs and immunized mice as a co-encapsulated vaccine product, as compared to two monovalent formulations dosed as separate injections. Equivalent, robust neutralizing antibody titers were observed regardless of formulation approach. The multivalency testing was expanded further to produce quadrivalent formulations, which were then administered to NHPs for immunization. These were compared to equivalent bivalent and monovalent vaccinations to assess the consistent robustness of the individual antigen immunogenicity. Robust neutralizing antibody titers were generated for all antigens across all conditions, supporting the potential of multivalent influenza vaccines as a viable approach without observations of immunodominance or interference of one antigen over another. Multi-antigen vaccines, if produced using traditional vaccine technologies, would require large manufacturing footprints utilizing antigen-specific operations; whereas an mRNA-based approach utilizes the same unit operations regardless of antigen produced, and therefore requires a much smaller footprint and infrastructure.

Our data indicate the suitability of the MRT platform for developing mRNA vaccines to multiple disease targets. We have optimized a generic manufacturing process, demonstrated in vivo expression of multivalent antigens with proper post-translational modifications that are necessary for a fully functional protein and the induction of adaptive responses that elicit both T and B cell immunity. To the best of our knowledge, this is an early report demonstrating the delivery of quadrivalent mRNA transcripts each coding for a distinct trimeric or tetrameric functionally active protein. We show that multiple mRNA transcripts can be packaged and delivered as a combination of two or four antigens with no differences in the magnitude of humoral immune responses compared to antigen delivered as a monovalent. Of particular utility is the ability to encapsulate multiple influenza virus antigens, as commonly envisioned for potential use in an influenza pandemic or a broad-spectrum seasonal vaccine, with improved manufacturing capacity for emergency scale-up.

High dose influenza vaccines have been demonstrated to provide protection beyond flu through reduction in hospitalizations due to influenza related cardiovascular events and pneumonia^57^. We have embarked into a journey to further improve influenza vaccine effectiveness through research and development across five pillars: antigen composition, adjuvants, effector mechanisms, host immunity and clinical design, and manufacturing platforms^58^. We address the last pillar in this work by building a platform that has a well-controlled scalable process and has shorter lead times allowing for facile applications to other antigens, making it pertinent for pandemic or seasonal viral vaccine production. Recognizing the broader consequences of influenza virus infection such as severity of disease and extent of morbidity and mortality is essential to determine the full burden of influenza^59^ and underscores the potential impact of the mRNA vaccine technology. Furthermore, the flexibility of the mRNA platform will allow inclusion of other viral targets, such as SARS-COV-2 antigens, should there be a need for such a seasonal vaccine in the future.

## Methods

### Ethics statement

All animal experiments were carried out in compliance with all pertinent US national institutes of health regulations and were conducted with approved animal protocols from the Institutional Animal Care and Use Committee (IACUC) at BIOQUAL Inc and New Iberia Research Center, University of Louisiana Lafayette (NIRC). Housing and handling of mice were performed in accordance and compliant with the standards of the Association for Assessment and Accreditation of Laboratory Animal Care (AAALAC), the Animal Welfare Act as amended, and the Public Health Service Policy. The studies adhered strictly to applicable Standard Operating Procedures of BIOQUAL Inc. Rockville, MD and the approved IACUC protocol. NHP studies were performed at the NIRC. Treatment of NHPs was in accordance with standard operating procedures at NIRCs, which adhere to the regulations outlined in the USDA Animal Welfare Act (9 CFR, Parts 1, 2, and 3) and the conditions specified in The Guide for Care and Use of Laboratory Animals (ILAR publication, 1996, National Academy Press.

### mRNA-LNP preparations

mRNA transcripts encoding for hEPO, FF, Cal09 HA, Sing16 HA, Mich15 NA, and Sing16 NA were synthesized by in vitro transcription employing RNA polymerase with a plasmid DNA template encoding the desired gene using unmodified nucleotides. The resulting purified precursor mRNA was reacted further via enzymatic addition of a 5’ cap structure (Cap 1) and a 3′ poly(A) tail of approximately 200 nucleotides in length as determined by gel electrophoresis and purified. All mRNA preparations were analyzed for purity, integrity, and percentage of cap1 before storage at -20 °C. Preparation of mRNA-LNP formulations were prepared by appropriate mixing solutions of mRNA and lipid mixture. Briefly, an ethanolic solution of a mixture of lipids (ionizable lipid, phosphatidylethanolamine, cholesterol and polyethylene glycol-lipid) at a fixed lipid and mRNA ratio were combined with an aqueous buffered solution of target mRNA at an acidic pH under controlled conditions to yield a suspension of uniform LNPs^60^. Upon ultrafiltration and diafiltration into a suitable diluent system, the resulting nanoparticle suspensions were diluted to final concentration, filtered, and stored frozen at -80 °C until use. For the multivalent formulations, co-encapsulation was performed by combining the mRNAs at the desired ratios and used as mRNA mix for the formulation process. The total mRNA encapsulated in the LNP was measured by the *RiboGreen*^*TM*^ assay and was used to calculate the representation of the different mRNAs used. An assumption was taken that the final ratio of mRNAs in LNP formulations is same as the starting ratios of mRNAs used. mRNA cap % measured using Agilent 1290 Infinity II UHPLC/ 6530 QTOF with MassHunter B.09.00 (Agilent Technologies). The mRNA-LNP formulations were characterized for size, percentage encapsulation and were stored at -80 °C at 1 mg/mL until further use by dilution with suitable buffer. LNP Size, polydispersity measurements were performed on Zetasizer 7.11 (Malvern) and % encapsulation was determined with Ribogreen assay measured using Spectramax M5 with SoftMax Pro 5.4 (Molecular Devices).

### Western blot

Human embryonic Kidney cells (HEK293FT ThermoFisher Cat# R70007) were cultured in DMEM (Gibco # D6429) contained 10% heat-inactivated FBS, 5 ml NEAA, and 1% G418 at 37 °C and 5% CO2 in a 6-well plate. Cells were then transfected with 3 µg of mRNA per well using TransIT®-293 Transfection Reagent (cat MIR 2225, Mirus**)** at 80% confluency. Twenty-four hours post transfection, cells were harvested and lysed with NP40 cell lysis buffer (cat FNN0021, Invitrogen). Lysate was subjected to SDS-PAGE and blotted on Nitrocellulose membrane with iBlot (IB30100, Invitrogen), and follow the manual of iBind and reagent (cat SLF1010 and SLF1020, Invitrogen) for western Blot. Cal09 HA protein was detected with anti-Cal09 recombinant HA Mouse polyclonal antibody (1:250 dilution, generated in house) followed by alkaline phosphatase-conjugated goat anti-mouse secondary antibody (1:1000 dilution, catalogue number A3562, Sigma). Similarly, Sing16 HA protein was detected with Anti-Sing16 H3 Rabbit polyclonal antibody Ab (1:250 dilution, inhouse reagent) followed by secondary antibody, goat anti-rabbit conjugated with alkaline phosphatase (1:1000 dilution, catalogue number ab97066, Abcam). Mich15 NA protein was detected with rabbit monoclonal antibody (1:250 dilution, catalogue number11058-r001, Sino Biologicals) followed by goat anti-rabbit conjugated with alkaline phosphatase (1:1000 dilution, ab97066, Abcam). Sing16 NA protein was detected with rabbit polyclonal antibody (1:250 dilution, catalogue number 40017-T60, Sino Biologicals) followed by goat anti-rabbit conjugated with alkaline phosphatase (1:1000 dilution, ab97066, Abcam).

### Visualization of HA and NA-proteins expressed in HeLa cells

Immunocytochemistry-immunofluorescence analysis of influenza NA and HA-proteins was performed in HeLa cells ATCC^Ⓡ^ CCL-2™ transfected with bivalent H3N2 (Sing16 HA and Perth09 NA) mRNAs LNPs)^30^. Glass coverslips were placed in each well of a 12-well plate and coated with 100 μg/mL solution of poly-D-lysine to aid in cell adhesion. HeLa cells were seeded at a density of 2 × 10e5 cells per well and allowed to grow overnight to achieve ≥80% cell confluency prior to transfection. Mirus TransIT-mRNA Transfection Kit (Mirus, cat# MIR2225) was used to transfect cells with Covid-19 Spike Protein mRNAs. In brief, per one well 1 μg of mRNA was added to 100 μL of Opti-MEM I Reduced-Serum Medium followed by 2 μL of mRNA Boost Reagent and 2 μL of TransIT-mRNA reagent. The mixture was incubated at room temperature for 5 min for the complexes to form. These complexes were then added dropwise to different areas of each well containing HeLa cells in 1 mL complete growth medium. Cells were incubated with the complexes for 24 hours (h) to permit expression of mRNAs. After 24 h cells were fixed in 4% paraformaldehyde and subjected to antibody staining for the HA or NA Proteins and an endoplasmic reticulum (ER) marker. After fixation cells were washed in phosphate buffer (PBS) followed by permeabilization in 0.5% Triton X-100 in PBS. Cells were then incubated for 0.3 h in a blocking solution consisting of 5% goat serum, 0.1% Triton X-100, 0.2% Bovine Serum Albumin, 50 mM ammonium chloride, 25 mM glycine, and 25 mM lysine in PBS. After washing with 0.1% Triton X-100 in PBS cells were incubated with primary antibodies against H3 HA (GeneTex GTX40258), NA (anti-Neuraminidase antibody, inhouse reagent), and ER marker Calnexin (Abcam ab22595) for 1 h at room temperature. Cells were then washed 3× in PBS containing 0.1% Triton X-100 followed by incubation for 1 h at room temperature with secondary antibodies. Goat anti-Mouse IgG Alexa Fluor 488 (ThermoFisher cat# A32723) and Goat anti-Rabbit IgG Alexa Fluor 555 (ThermoFisher, cat# A32732) secondary antibodies were used at 4 μg/mL concentrations along with DAPI for visualization of nuclei. After incubation cells were washed 3 times in PBS containing 0.1% Triton X-100. Coverslips were then removed from wells and placed on glass slides with a drop of Aqua-Poly/Mount mounting medium (Polysciences, cat# 18606). After sealing the coverslips with nail polish, and images were captured on Zeiss LSM880 confocal microscope followed by image analysis for quantification of HA and NA colocalization to the ER, mean signal intensity, and percent of cell area. Image analysis was performed with Visiopharm image analysis software where mask layers were overlayed on HA, NA, and ER immunofluorescent staining based on HA or NA colocalization with ER marker. HA or NA staining which colocalized with the ER were highlighted in yellow and HA or NA staining which did not colocalize with ER were highlighted in green.

### Flow cytometry

Human skeletal muscle cells (HskMCs, Lonza, Cat #: CC-2561) were cultured in M199 (Life Technologies) supplemented with GlutaMax (Life Technologies), streptomycin, penicillin (Gibco), and 20% heat inactivated FBS (VWR) at 37 °C with 5% CO_2_. The cells were harvested by trypsinization, washed with PBS, and electroporated using human primary muscle cell transfection kit on Nucleofector 2b (Lonza) with 12 mg of mRNA per 10^6^ cells following manufacturer’s electroporation program D-033. Post 24 hr harvested cells were fixed, permeabilized with CytoFix/Perm (BD) and stained with Cal09 HA (Immune Tech), Sing16 HA (30-2F11-F7-A5, GeneTex), Mich15 NA (6G6, Immune Tech) and Sing16 NA (40017-RP01, Sino Biologicals) specific Ab followed by PE conjugated goat anti-mouse IgG secondary Ab (Southern Biotech) or AF647 conjugated goat anti-rabbit IgG (Life Technologies). Then the Ab labeled cells were acquired by Fortessa (BD) and the expression of each protein was analyzed by FlowJo 10.6.2 (BD, Ashland, OR). Blots for each antigen were processed in the same experiment and were processed in parallel.

### Cryogenic transmission electron microscopy

A PELCO easiGlow device was used to plasma clean the grids prior to LNP sample application, and a Vitrobot Mark IV System (ThermoFisher) with the chamber held at 100% humidity and 18 °C was used for plunge freezing. A 3.0 µL droplet of LNP sample was dispensed onto 300 mesh R2/1 quantifoil grids with carbon film and gold bars. Grids were blotted for 4 seconds, held in place for 10 s, and then immediately plunge frozen in liquid ethane for storage and transfer to a Krios microscope. Exposures were collected using a Titan Krios transmission electron microscope (ThermoFisher) equipped with a BioQuantum energy filter and K3 direct electron detector (Gatan) operating in counting mode. Calibrated physical pixel size at the detector was 1.38 Å, corresponding to ×64,000 magnification. A total of 3141 69-frame movie exposures were collected at a dose per frame of 1.045 e/Å2 with defocus between -0.5 and -1.7 um. For each movie exposure, patch-based motion correction, binning of super-resolution pixels, and frame dose-weighting was performed using RELION-3.1.34. From corrected images, over 700 candidate particle coordinates were extracted. Subsequent data analysis was done with Matlab R2019a with image processing toolbox.

### Immunization of mice and NHPs

For expression studies groups of four *cynomolgus* macaques (NHPs) (male and female) and four to eight male BALB/c mice were administered intramuscularly either dose of 10 µg (NHP) or 1, 0.5, 0.1, 0.05 µg (mice) with hEPO-LNP prepared in the same ratio as the one intended to be used for HA/NA mRNA-LNP formulations. Blood samples depending on the species were taken pre-administration, and at 6 h, 24 h, 48 h, 72 h, or 96 h post administration to monitor for serum hEPO expression via an ELISA using R and D Systems, Quantikine® IVD® ELISA, Human Erythropoietin Immunoassay kit as per manufacturers protocol. and reported as final values of mIU/mL and ng/mL. Briefly, microplate wells, precoated with a mouse monoclonal antibody specific for EPO were incubated with specimen or standard. After removing excess specimen or standard, wells are incubated with a rabbit anti-EPO polyclonal antibody conjugated to horseradish peroxidase. During the second incubation, the antibody-enzyme conjugate binds to the immobilized EPO. Excess conjugate is removed by washing. A chromogen is added to the wells and is oxidized by the enzyme reaction to form a blue colored complex. The reaction is stopped by the addition of acid, which turns the blue to yellow. The amount of color generated is directly proportional to the amount of conjugate bound to the EPO antibody complex, which, in turn, is directly proportional to the amount of EPO in the specimen or standard. The absorbance of this complex is measured, and a standard curve is generated by plotting absorbance versus the concentration of the EPO standards. The EPO concentration of the unknown specimen is determined by comparing the optical density of the specimen to the standard curve. The standards used in this assay are recombinant human EPO calibrated against the Second International Reference Preparation (67/343), a urine-derived form of human erythropoietin. FF-LNPs were utilized to check level of expression of target protein in vivo using imaging on IVIS® Lumina LT In Vivo Imaging System with Living image 4.0 (PerkinElmer).

For immunogenicity studies groups of 8 female Balb/c mice (*Mus musculus*) per treatment group were immunized under isoflurane anesthesia with a dose of 0.05 mL of designated vaccine preparation or diluent via the IM route in the quadriceps, on day 0 in one hind leg and day 28 in the contralateral leg. Mice that lost more than 20% of their initial body weight and displayed severe clinical signs were euthanized after the veterinarian’s assessment of the animal’s health prior to the study termination.

Naive male and female Mauritius origin Cynomolgus macaques (*Macaca fascicularis*) were selected from the Sanofi NHP colony at New Iberia Research Center. Animals weighed >2 kg and were >2 years of age at the start of the studies. Animals selected for the study underwent comprehensive physical examinations prior to assignment to the study. The pre-assignment assessment of health status included a hands-on veterinarian examination and blood sample collections for CBC analysis as applicable per NIRC SOPs. Animals were generally housed in pairs and acclimated for at least 3 days prior to the start of the study. Groups consisted of up to 6 animals per treatment group. All animals were immunized under ketamine HCl (10 mg/kg, IM) or telazol (4–8 mg/kg, IM) sedation with a dose of 0.5 mL of their respected vaccine preparation or diluent via the IM route in one forelimb of each animal, targeting the deltoid, on Study Day 0. Twenty-eight days after the first immunization took place, a second immunization was given to the animals in the contralateral limb.

For challenge studies, mice were inoculated with the challenge strain approximately 9–12 weeks after the last immunization. Vials of stock virus were thawed and diluted to the appropriate concentration in ice-cold sterile PBS. All mice were challenged with a total volume of 50 μL containing 105.54 TCID_50_ of A/Belgium/145-MA/2009 virus in PBS which equated to 4LD_50_. Virus challenge was performed inside the biosafety cabinet in an enhanced ABSL2 laboratory. Mice were first anesthetized with an IP injection of a Ketamine/Xylazine solution (50 mg/kg Ketamine and 5 mg/kg Xylazine), and then challenged IN (dropwise into both nostrils; 25 μL per nostril) with a total volume of 50 μL of influenza virus using a micropipette. Following the challenge procedure, mice were placed in dorsal recumbency and observed until recovery from anesthesia. Daily body weights were taken following H1N1 challenge. Any individual animal with a single observation > 20% body weight loss was euthanized. The weight measurements were either recorded daily post challenge until euthanasia in the online database, Pristima® (Version 7.5.0 Build 8), or written on study specific working sheets.

### Blood collection

Blood was collected from mice via submandibular or orbital sinus bleeds (in-life bleed, pre-study and on study days 14, 28, and 42 approximately 200 μL) and cardiac puncture (terminal bleed, day 56) from all animals under sedation. Mice were bled on pre-study to obtain a base-line pre-immune serum sample and for pre-screening purposes. Processing of the serum, blood samples were collected into SST tubes and allowed to clot for 30 min to 1 h at room temperature. The samples were then centrifuged 1000–1300 g for 5–10 min with brakes off. Serum was collected using a P200 pipettor, divided into two 0.5 mL cryovials, and stored at −20 °C. All bleeds were documented on specimen collection and processing logs, indicating the time of sample collection and the technician responsible for performing the procedure. A portion of the serum samples were evaluated in the HAI or ELLA and ELISA assays for antibody titers.

NHPs were bled for serum isolation while under anesthesia administered intramuscularly using10 mg/kg ketamine/1 mg/kg Acepromazine (days -4, 2, 7, 14, 28, 30, 35, 42, 56, 90, 180). The volume of blood withdrawn did not exceed established guidelines with respect to percentage of body weight and animal’s physical condition Blood was withdrawn from anesthetized NHPs using femoral venipuncture using a Vacutainer 21 ga × 1″ blood collection needle or Abbott Butterfly 23 ga x ¾” tubing attached to BD Vacutainer® SST™ gel tubes. Serum was isolated by spinning the tubes at room temperature at a speed of 1200 × *g* for 10 min. Serum will then be aliquoted into labeled cryovials (1 mL/vial) and stored at ≤ -20 °C. A portion of the serum samples were evaluated in the HAI or ELLA and ELISA assays for antibody titers. For PBMCs, NHPs were pre-bled before vaccination and again approximately 42–63 days after the first injection. For this purpose, blood is collected into BD Vacutainer® tubes containing heparin anticoagulant. Briefly, anticoagulated blood samples are diluted in PBS and subjected to gradient density centrifugation for 30 min at 400 × *g* using Histopaque separation solution (Sigma, St. Louis, Mo.). The opaque interface containing mononuclear cells is then collected, washed three times in PBS using a low speed (250 × *g*) centrifugation to reduce the number of platelets. The live vs. dead PBMC are enumerated using a Nexcelom Cellometer K2. The PBMC are cryopreserved in FBS with 10% DMSO using Mr. Frosty® freezing boxes. The boxes are placed immediately into a -80 °C freezer for 24 h and then transferred for storage in a liquid nitrogen tank.

### ELISA

The antibody ELISAs were performed using recombinantly produced influenza A H3N2 (Sing16) Neuraminidase (NA) protein, influenza A H3N2 (Sing16) hemagglutinin (HA) protein, or influenza A H1N1 (A/California/07/2009) hemagglutinin (HA) protein. The proteins were captured on 96 well high binding polystyrene plates at a concentration of 2 μg/mL in Carbonate-Bicarbonate Buffer. The plates were covered and incubated overnight (16 ± 4 h) at 2–8 °C. After overnight incubation, the antigen coated plates were washed 5 times with Washing Buffer (PBS 0.5% Tween20) and blocked with Blocking solution (10% BSA in PBS) for 60 ± 30 min at room temperature. Test samples, naive control, and the reference sample were diluted in Sample Diluent (PBS 10% BSA 0.5% Tween 20) and added to wells in duplicates followed by incubation at room temperature for 90 min. Plates were washed 5 times with Washing Buffer, and Goat anti-mouse HRP for mouse sera or Goat anti-monkey HRP for NHP sera was added at a dilution of 1:10,000. The plates were then incubated 30 min at room temperature and the excess HRP-IgG was washed with Washing buffer. Sure-Blue TMB substrate was added to each plate and the reaction was stopped after ~10 min with TMB stop solution. The plates were then read at 450 nm with a Thermo Labsystems Multiskan spectrophotometer. The anti-antigen (HA or NA) specific antibody titers were expressed as a reciprocal of the highest serum dilution with an absorbance value >0.3.

### HAI assay

HAI assays were performed using the A/Singapore/INFIMH-16-0019/2016 (H3N2) and the A/California/07/2009 (H1N1) virus stocks from BIOQUAL, Inc. Sera were treated with receptor-destroying enzyme (RDE) by diluting one-part serum with three parts enzyme and incubated overnight in a 37 °C water bath. Enzyme was inactivated by a 30-minute incubation period at 56 °C followed by addition of six parts PBS for a final dilution of 1/10. HAI assays were performed in V-bottom 96-well plates using four hemagglutinating units (HAU) of virus and 0.5% turkey RBC. The reference serum for each strain was included as a positive control on every assay plate. Each plate also included a back-titration to confirm the antigen dose (4 HAU/25 μL) as well as a negative control sample (PBS or naive control serum). The HAI titer was determined as the highest dilution of serum resulting in complete inhibition of hemagglutination. Results were only valid for plates with the appropriate back-titration result (verifying 4 HAU/25 μL added) and a reference serum titer within 2-fold of the expected titer.

### NAI assay

The method for the Enzyme-Linked Lectin Assay (ELLA) assay was used to determine Neuraminidase-Inhibiting antibody titers. The source of antigen (NA) was titrated, and a standard amount was selected for incubation with serial dilutions of serum. Virus was used as the source of NA in all cases. Titration of sera was performed with serial dilutions of sera (heat inactivated at 56 °C for 1 h) and a standard amount of virus was added to duplicate wells of a fetuin-coated plate. This mixture was then incubated overnight (16–18 h); the next day, HRP conjugated Peanut Agglutinin PNA (diluted to 2.5 μg/mL) was added to the washed plate and incubated for 2 h at room temperature. Substrate (ODP in Sodium citrate) was added and incubated for 10 min to develop the color. Next Stop buffer (1 N sulfuric acid) was added to stop the reaction. Plates were scanned for absorbance at OD 490 nm. The reduction or absence of color relative to a viral control indicated inhibition of NA activity due to the presence of NA-specific antibodies. NAI titers (IC_50_ values) were calculated from the OD readings and the results were graphed in GraphPad Prism. If ELLA titration curves did not allow a good fit to determine a reliable IC_50_ value, the samples were retested using a different dilution scheme to reach the 50% endpoint.

### T cell ELISPOT Assay

Complete medium (DMEM1640 + 10% heat-inactivated FCS) was prewarmed in a 37 °C water bath. PBMCs were quickly thawed in a 37 °C water bath and transferred dropwise to conical tubes with the prewarmed medium. The tubes were centrifuged for 5 mins at 433 g in Allegra X-30R centrifuge (rotor SX4400, Beckman Coulter, Inc) and the cells were resuspended and counted using a Guava cell counter. Monkey IFNγ ELISPOT kit (Mabtech 3421M-4APW) and IL-13 ELISPOT kit (Mabtech 3470M-4APW) were used. Precoated plates provided by the kits were washed four times with sterile PBS and blocked with 200 µL of complete medium in 37 °C incubator for at least 30 min. A/SINGAPORE/INFIMH160019/2016 H3 peptides pool (Genscript Custom Order) (at 1 µg/mL of each peptide) were used as recall antigens in the assay. Two µg/mL of ConA (Sigma CAT#C5275) was used as a positive control. Fifty µL of recall antigens and 300,000 of PBMCs in 50 µL were added to each well for stimulation. The plates were placed in a 37 °C, 5% CO_2_ humidified incubator for 48 h.

After the incubation, cells were removed, plates were washed 5 times with PBS, and 100 µL of 1 µg/mL biotinylated anti-IFNγ or anti-IL-13 detection antibodies were added to each well in the plates. After 2 hr incubation, the plates were washed 5 times with PBS and incubated with 100 µL of a 1:1000 dilution of streptavidin in each well for one hour at room temperature. Plates were developed with 100 µL of BCIP/NBT substrate solution until the spots emerged. Plates were rinsed by tap water, air-dried and scanned and counted using CTL ImmunoSpot Reader (Cellular Technology Ltd). T The data was reported as spots forming cells (SFC) per million PBMCs.

### Memory B cell (MBC) ELISPOT assay

Human IgG Single-Color memory B cell ELISPOT kit (CAT# NC1911372, CTL) was used per manufacturer’s instruction to measure A/Singapore/16/H3-specific and total IgG+ antibody-secreting cells (ASCs). Differentiation of MBCs into ASCs was performed in PBMC using a stimulation cocktail provided by the kit. Briefly, frozen PBMCs were quickly thawed in a 37 °C water bath, mixed with DNase 1 (CAT# 90083, FISHER SCIENTIFIC) and transferred into the tube containing pre-warmed complete culture medium (CM) (RPMI 1640, (CAT# 22400-089, gibco) containing 10% FCS (CAT # SH30073.03, HyClone), and 1% penicillin/streptomycin (CAT# P4333, Sigma)) for 5 minutes at 433 g in Allegra X-30R centrifuge (rotor SX4400, Beckman Coulter, Inc). Cell pellet was re-suspended in 5 ml of complete medium at 2 × 10(6) cells per ml and transferred to a T25 flask for 1 h in 5% CO_2_ incubator at 37 °C. The volume of cell suspension was then adjusted to 6 ml and B-Poly-S was added at 1:1000 dilution. Cells were left in CO2 incubator for stimulation for 4 days. PVDF microplates supplied by the kit were pre-wetted with 70% ethanol, rinsed and coated overnight with 80ul/well of either anti-human IgG capture Ab provided by the kit or A/Singapore/16/H3 recombinant protein at 4 μg/ml.

Cells were harvested after 4 days of stimulation, washed, and counted and adjusted to the designated concentration in the CM. Coated microplates were washed with PBS, blocked for 1 h with the CM and emptied out. Cell suspension at 100 µl/well was added to the plates and incubated in CO_2_ incubator at 37 C for 18 h. After washing, 80 µl/well of 1:400 diluted anti-human IgG Biotin detection antibody was added to the plate and incubated at RT for 2 h. Following washing, Streptavidin-AP at 1:1000 dilution was added to the plate at 80 ul/well for 1 h. Freshly prepared Substrate solution was added and incubated at RT for 18 min. Plates were rinsed by tap water, air-dried and scanned and counted using CTL ImmunoSpot Reader (Cellular Technology Ltd). For each individual animal the number of IgG+ and number of A/Singapore/16/H3-specific ASCs was calculated per million of PBMCs. The frequency of antigen-specific ASCs was calculated as % of antigen-specific ASCs to the total IgG+ ASCs. To assess assay background the negative control wells on every plate were coated with PBS (no background was detected).

### Statistical analysis

For estimating the Tmax of Radiance, non-parametric method was used to estimate the Tmax of individual subject based on observed data. For estimating the half-life of Radiance, assuming exponential decay model for radiance after reaching the maximum value, linear model was fitted to log transformed data per subject during the time course from the maximum radiance to decay to baseline (we estimate the baseline using the average of radiance in saline group). The half-life was estimated as the time point when the log radiance had reached the middle point between maximum and baseline values. For analysis of different readouts with results summarized as geometric mean and SE model based geometric means and SEs were estimated from mixed effect model for repeated measures where the response was the log transformed readouts, vaccination was fixed effect and time was repeated measure; log-based means and SE estimates from the model were then back transformed to get geometric means and SEs. For weight change over time, descriptive statistical analysis was used. Medians and ranges of each group of the maximum %body weight loss from baseline (day 0) over time were reported to evaluate the worse scenarios; medians and ranges of each group of the %body weight change from baseline at the last observation were reported to evaluate the body weight recovery. Statistical analyses were performed using SAS version 9.4. All statistical tests were two-sided with significant level of 0.05.

### Reporting summary

Further information on research design is available in the Nature Research Reporting Summary linked to this article.

## Supplementary information

Supplementary Information

Reporting Summary

## Data Availability

The datasets generated during and/or analyzed during the current study are available from the corresponding author on reasonable request.
