# Supplementary material for: Development of multivalent mRNA vaccine candidates for seasonal or pandemic influenza
Source: NPJ Vaccines. 2021 Dec 16;6:153. doi: 10.1038/s41541-021-00420-6 (PMC8677760; doi:10.1038/s41541-021-00420-6)
Supplement: Supplementary file 1 — Supplementary Information [file 41541_2021_420_MOESM1_ESM.pdf]

Supplementary Data

Supplementary Figure 1: Characterization of m RNA–LNP.

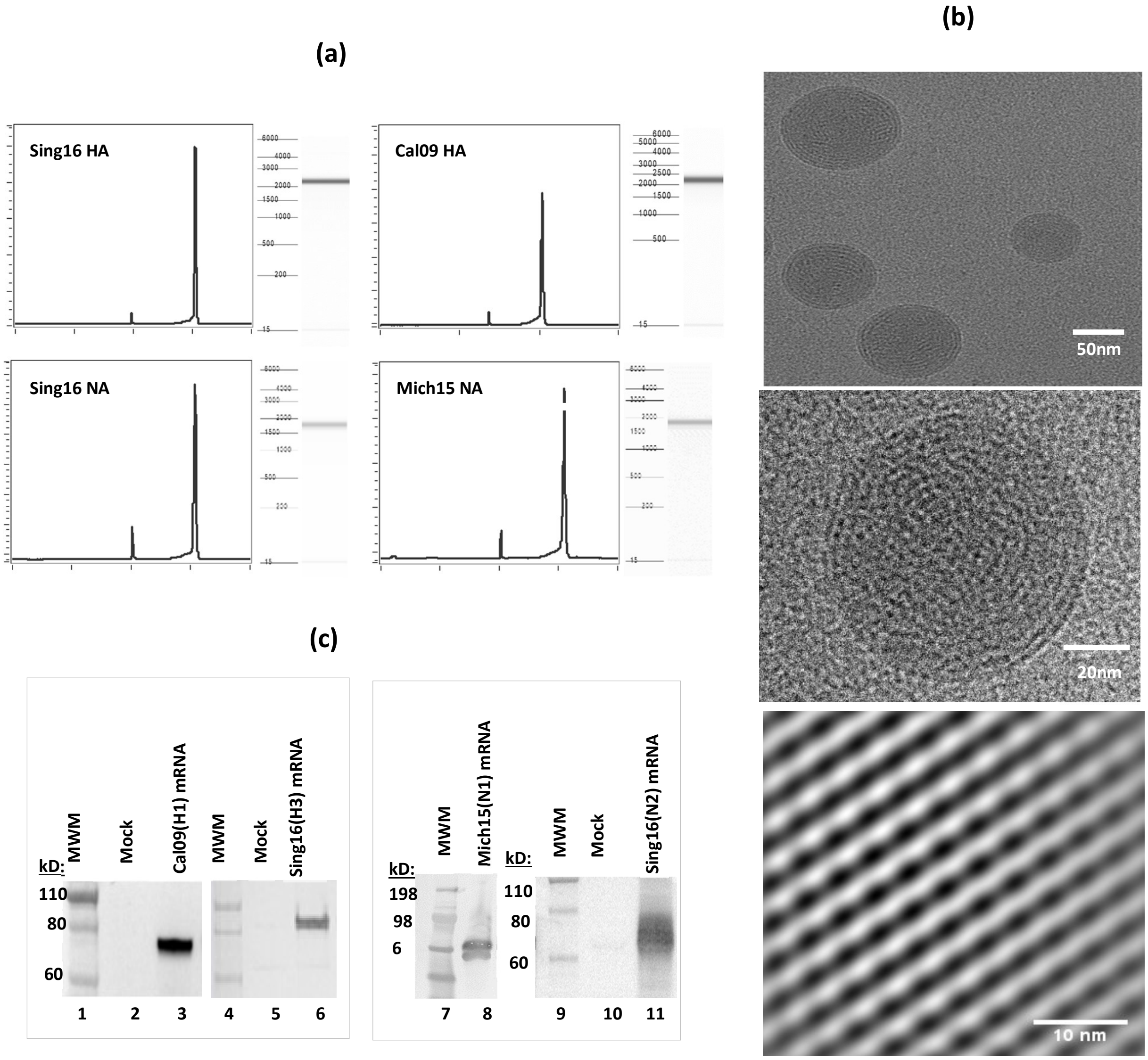

(a) Integrity of purified cap and tailed mRNA by capillary electrophoresis **(b)** Cryo-EM images of Cal09 mRNA-LNP showing spherical (left) multi-lamella inner core structure (middle). The solid core structure showing periodicity of layer, when analyzed by Fourier Transform (right). The representative scale bar in the left, middle and right panel is equivalent to 50nm, 20nm and 10nm respectively. **(c)** *In vitro* expression of HA and NA in HEK293FT

cells transfected with mRNA by western blot analysis. Cell lysates from transfected HEK293FT cells: lane 3 Cal09(H1) HA mRNA and analyzed with Anti-Cal09 H1 Mouse polyclonal antibody (internally generated); lane 6 Sing16(H3) HA mRNA and analyzed with Anti-Sing16 H3 Rabbit polyclonal antibody (internally generated); lane 8 Mich15 NA mRNA and analyzed with 11058-r001, Rabbit monoclonal antibody from Sino Bio; and lane 11 Sing16 NA mRNA and analyzed with Rabbit anti-N2 polyclonal, cat 40017-T60, Sino Biologicals. Molecular weight markers (MWM) are lanes 1, 4, 7, 9 and mock transfected cells are lanes 2, 5 & 10.

Supplementary Figure 2: Gating strategy for expression studies in muscle cells.

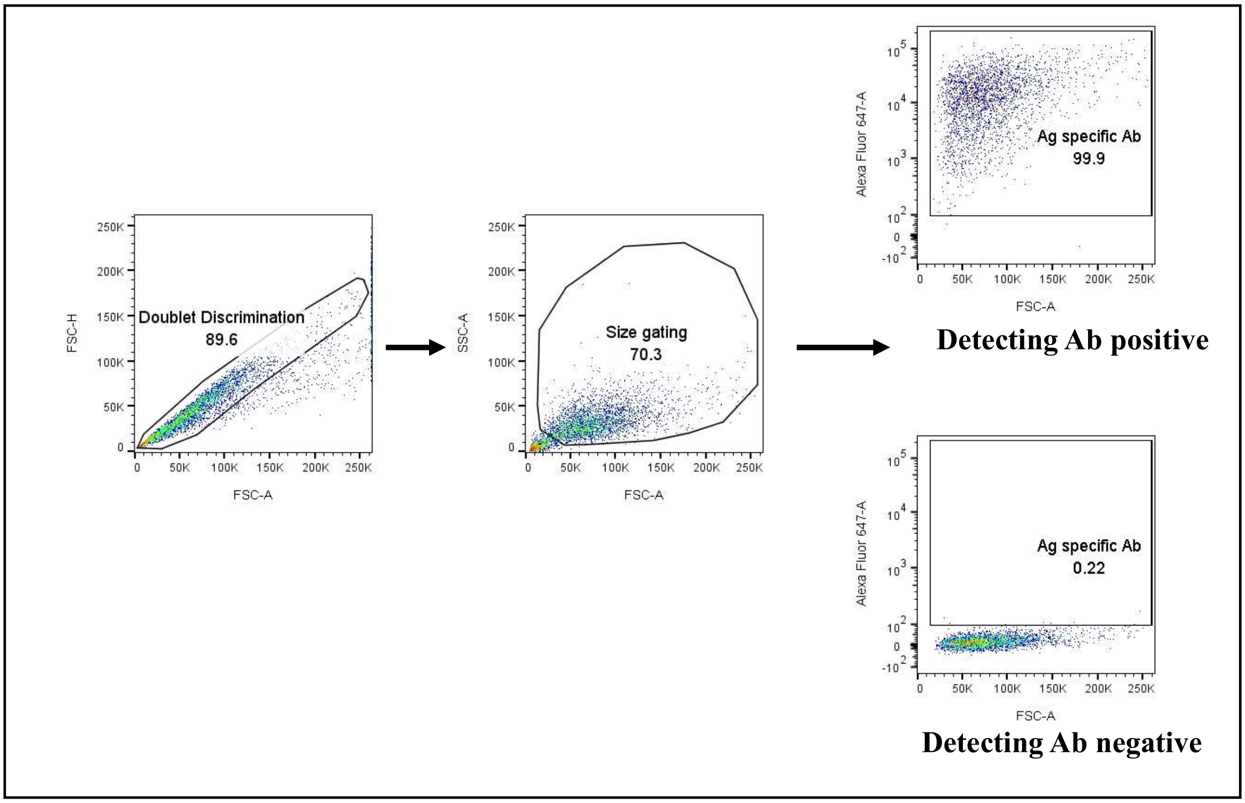

Gating strategy for HskMC flow cytometry. HskMC cell events acquired by flow cytometer were gated for doublet discrimination by FSC-A and FSC-H, then gated by proper cell size by FSC-A and SSC-A. Antigen specific population gate was determined by mock treated HskMC as negative control (Ag; antigen; Ab; antibody).

Supplementary Figure 3 Functional Verification of mRNA-LNP Formulations.

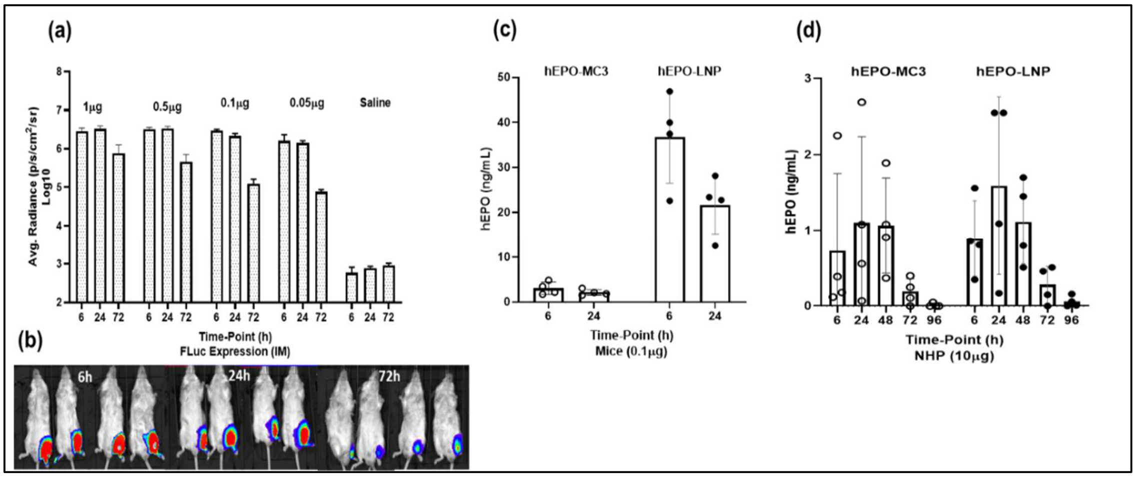

(a) Expression of luciferase (FF) in BALB/c mice. A single dose of FF mRNA-LNP (5, 1, 0.1, 0.05 μg) was injected in mice (n=4) by IM route. Luciferin 3 mg was injected at the time of whole animal imaging using IVIS Spectrum, Perkin Elmer recording bioluminescence

intensity. Images of whole animal average radiance at 6, 24, and 72h after injection. Radiance recorded for 1, 0.5, 0.1 and 0.05 μg dose administrations of Luc mRNA-LNP are shown in graph. Bars represent mean with standard deviation **(b)**Whole animal images indicating total flux of luminescence, at 6 to 72h. Total flux of luminescence in groups of mice (n=4) receiving 0.1 μg dose of FF-LNP are shown. **(c)** Expression of hEPO in BALB/c mice. A single dose of hEPO mRNA-LNP (0.1 μg) was injected in BALB/c mice by IM route. hEPO expression was quantified in serum at 6h and 24h after administration using ELISA. Bars represent means and standard deviations. **(d)** Expression of hEPO in NHP. A single dose of hEPO mRNA-LNP (10 μg) was injected in Cynomolgus macaques by IM route. hEPO expression was quantified in serum at 6, 24, 48, 72, & 96h after administration, using ELISA. Each dot represents an animal and bars represent means and standard deviations.

Supplementary Figure 4: Serological Evaluation of HA mRNA-LNP in mice.

(a) (b)

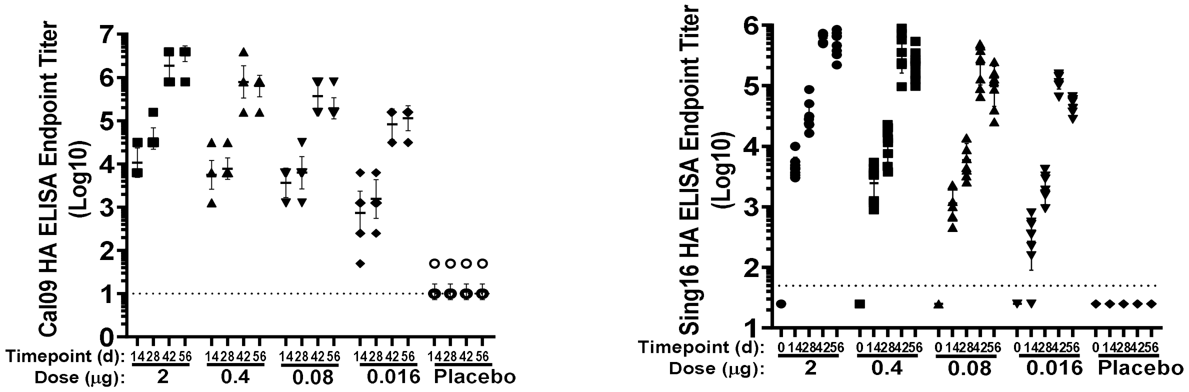

BALB/c mice (n=8 per group) were immunized twice IM, 4 weeks apart with 2, 0.4, 0.08, and 0.016 µg of either Cal09 HA mRNA-LNP or Sing16 HA mRNA-LNP. ELISA titers recorded for sera collected at days 14, 28, 42, 56 against **(a)** A/California/07/2009 H1N1 influenza virus recombinant HA **(b)** A/Singapore/INFIMH160019/2016 H3N2 influenza virus recombinant HA are shown. Each dot represents an individual animal, and the line represents the geometric mean for the group. Lower horizontal line in each panel represents the lower limit of assay read out.

Supplementary Figure 5: Serological Evaluation of NA mRNA-LNP in mice.

(a) (b)

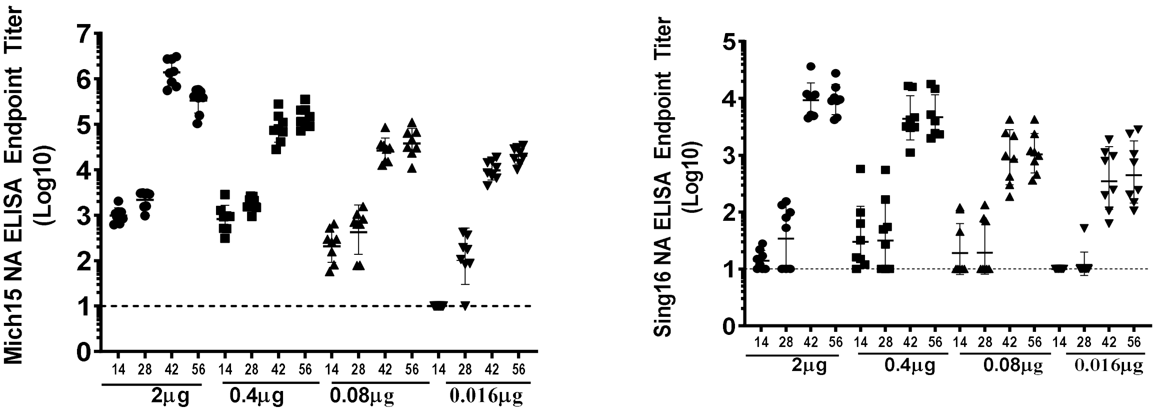

BALB/c mice (n=8 per group) were immunized twice IM 4 weeks apart with 2, 0.4, 0.08, and 0.016 µg of either Mich15 NA mRNA-LNP or Sing16 NA mRNA-LNP. Total IgG titers recorded for sera collected at days 14, 28, 42, 56 against **(a)** A/Michigan/45/2015 (N1 influenza virus recombinant NA **(b)** A/Singapore/INFIMH160019/2016 (N2) virus recombinant NA are depicted. Each dot represents an individual animal, and the line represents the geometric mean for the group. Lower horizontal line in each panel represents the lower limit of assay read out.

Supplementary Figure 6: Serological Evaluation of HA Sing16 HA mRNA-LNP in NHP.

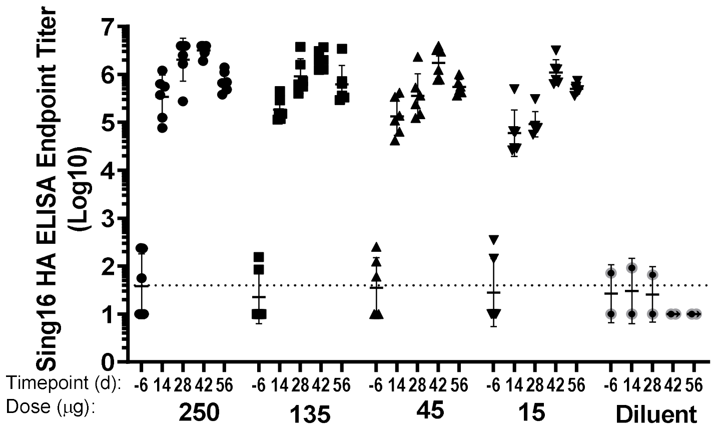

Cynomolgus macaques (n=6 per group) were injected twice, 4 weeks apart by IM route, with 15, 45, 135 or 250 µg of Sing16 HA mRNA-LNP. Serum samples were collected at days -6, 14, 28, 42, and 56. Log 10 IgG titers against recombinant HA protein of A/Singapore/INFIMH160019/2016 virus are shown. Each dot represents an individual animal, and the line represents the geometric mean for the group. Lower horizontal line represents the lower limit of assay read out.

Supplementary Table 1: Attributes of LNP formulations used in mouse preclinical testing.

LNP	Size (nm)	PDI	% Encapsulation
**Cal09 HA**	97.54	0.117	95.2
**Sing16 HA**	103.2	0.068	97.3
**Sing16 NA**	105.8	0.128	96.5
**Mich15 NA**	103.3	0.136	97.4

Size and PDI of LNP measured using dynamic light scattering and % encapsulation using RiboGreen™ assay.

Supplementary Table 2: Statistical analysis of HAI and ELISA titers in NHPs vaccinated with H3 mRNA-LNP

Time Point	Dose effect of Sing16HA mRNA-LNP (ELISA)	Dose effect of Sing16HA mRNA-LNP (HAI)	Dose effect of Sing16HA mRNA-LNP (MN)
	p-value	p-value	
Day 14	p=0.03	p=0.2711	p=0.5759
Day 28	p<0.001,	p=0.0359	p=0.02
Day 42	p=0.0251	p=0.2178	p=0.4126
Day 56	p=0.7095	p=0.174	p=0.1938

Mixed effect model for repeated measures with the dose, time and their interaction as fixed factor. Time was included as repeated measures. The first part consisted in verifying at which time point a statistically significant difference was observed between the doses

Time Point	Pair wise dose comparison of Sing16 HA mRNA-LNP (ELISA)			
			Fold (95% CI)	p-value
Day 28	15 µg	45 µg	/3.8 (1.3-11.4)	0.0181
	15 µg	250 µg	/22.0 (7.4-65.5)	<0.001
	135 µg	15 µg	x10.0 (3.4-29.7)	<0.001
	135 µg	45 µg	x2.6 (0.9-7.7)	0.0826
	135 µg	250 µg	/2.2 (0.7-6.6)	0.1464
	250 µg	45 µg	x5.7 (1.9-17.1)	0.0033
Day 42	15 µg	45 µg	/1.6 (0.8-3.1)	0.1636
	15 µg	250 µg	/2.9 (1.5-5.6)	0.0031
	135 µg	15 µg	x1.8 (1.0-3.6)	0.0670
	135 µg	45 µg	x1.2 (0.6-2.3)	0.6289
	135 µg	250 µg	/1.6 (0.8-3.0)	0.1688
	250 µg	45 µg	x1.8 (0.9-3.6)	0.0694
**Time Point**	**Pair wise dose comparison of Sing16 HA mRNA-LNP (HAI)**			
			**Fold (95% CI)**	**p-value**
Day 28	15 µg	45 µg	/4.0 (0.9-17.2)	0.0618
	15 µg	135 µg	/9.0 (2.1-38.7)	0.0051
	15 µg	250 µg	/4.5 (1.0-19.4)	0.0445
	45 µg	135 µg	/2.2 (0.5-9.7)	0.2628
	45 µg	250 µg	/1.1 (0.3-4.8)	0.871
	135 µg	250 µg	x2.0 (0.5-8.6)	0.3352
Day 42	15 µg	45 µg	/1.8 (0.8-4.0)	0.1483
	15 µg	135 µg	/2.2 (1.0-5.0)	0.0479
	15 µg	250 µg	/1.4 (0.6-3.1)	0.3781
	45 µg	135 µg	/1.3 (0.6-2.8)	0.5547
	45 µg	250 µg	x1.3 (0.6-2.8)	0.5547
	135 µg	250 µg	x1.6 (0.7-3.5)	0.2433
**Time Point**	**Pair wise dose comparison of Sing16 HA mRNA-LNP (MN)**			

			Fold (95% CI)	p-value
Day 28	15 µg	45 µg	/4.5 (1.2-17.4)	0.0315
	15 µg	135 µg	/9.0 (2.3-34.8)	0.0029
	15 µg	250 µg	/2.5 (0.7-9.8)	0.1707
	45 µg	135 µg	/2.0 (0.5-7.8)	0.2994
	45 µg	250 µg	x1.8 (0.5-6.9)	0.3853
	135 µg	250 µg	x3.6 (0.9-13.8)	0.0646
Day 42	15 µg	45 µg	/1.9 (0.8-4.3)	0.1389
	15 µg	135 µg	/1.8 (0.7-4.2)	0.1812
	15 µg	250 µg	/1.6 (0.7-3.8)	0.2336
	45 µg	135 µg	x1.0 (0.4-2.5)	0.9193)
	45 µg	250 µg	x1.1 (0.5-2.6)	0.7582
	135 µg	250 µg	x1.1 (0.5-2.6)	0.8445

*Mixed effect model for repeated measures with the dose, time and their interaction as fixed factor. Time was included as repeated measures. Detailed pairwise comparisons show the fold differences (+95% CI) associated with their p-value***.**

HAI Titers: Difference in boost effect for all groups between day 28 and day 42.

**Sing16 HA mRNA-LNP**	**Boost effect**		**Results**	
			**Fold (95% CI)**	**p-value**
15 µg Sing16 HA mRNA-LNP	D28	D42	**/14.3 (7.7-26.4)**	**p<0.001,(S)**
45 µg Sing16 HA mRNA-LNP	D28	D42	**/6.3 (3.4-11.7)**	**p<0.001,(S)**
135 µg Sing16 HA mRNA-LNP	D28	D42	**/3.6 (1.9-6.6)**	**p<0.001,(S)**
250 µg Sing16 HA mRNA-LNP	D28	D42	**/4.5 (2.4-8.3)**	**p<0.001,(S)**

ELISA Titers: Difference in boost effect for all groups between day 28 and day 42.

**Sing16 HA mRNA-LNP**	**Boost effect**		**Results**	
			**Fold (95% CI)**	**p-value**
15 µg Sing16 HA mRNA-LNP	D28	D42	**/11.9 (5.9-24.1)**	**p<0.001,(S)**
45 µg Sing16 HA mRNA-LNP	D28	D42	**/4.9 (2.4-9.9)**	**p<0.001,(S)**
135 µg Sing16 HA mRNA-LNP	D28	D42	**/2.2 (1.1-4.4)**	**p=0.027,(S)**
250 µg Sing16 HA mRNA-LNP	D28	D42	/1.6 (0.8-3.2)	p=0.2007,(NS)

MN Titers: Difference in boost effect for all groups between day 28 and day 42.

**Sing16 HA mRNA-LNP**	**Boost effect**		**Results**	
			**Fold (95% CI)**	**p-value**
15 µg Sing16 HA mRNA-LNP	D28	D42	**/12.7 (6.3-25.6)**	**p<0.001,(S)**
45 µg Sing16 HA mRNA-LNP	D28	D42	**/5.3 (2.6-10.6)**	**p<0.001,(S)**
135 µg Sing16 HA mRNA-LNP	D28	D42	**/2.5 (1.2-5.3)**	**p=0.0166,(S)**
250 µg Sing16 HA mRNA-LNP	D28	D42	**/8.3 (4.1-16.6)**	**p<0.001,(S)**

Supplementary Table 3: Frequency of antigen-specific memory B cells in NHP vaccinated with H3 mRNA-LNP

Animal group	Animal ID	PBMCs/well of Ag-specific IgG	Spot # of Ag-specific IgG/million PBMCs	PBMCs/well of total IgG	Spot # of total IgG/million PBMCs	% of Ag-specific IgG to total IgG
**H3 mRNA-LNP (45 µg)**	1	3×10^5	1082	5×10^3	21700	5.0
	2	3×10^5	232	5×10^3	6100	3.8
	3	3×10^5	282	5×10^3	11700	2.4
	4	3×10^5	2	5×10^3	100	2.0
	5	3×10^5	283	5×10^3	8700	3.3
	6	3×10^5	225	5×10^3	22800	1.0
**H3 mRNA-LNP (15 µg)**	1	3×10^5	63	5×10^3	21600	0.3
	2	3×10^5	58	5×10^3	11300	0.5
	3	3×10^5	253	5×10^3	17300	1.5
	4	3×10^5	173	5×10^3	17300	1.0
	5	3×10^5	63	5×10^3	9300	0.7
	6	3×10^5	107	5×10^3	19300	0.6
**rHA (45 µg)**	1	3×10^5	2	5×10^3	19800	0.0
	2	3×10^5	28	5×10^3	14300	0.2
	3	3×10^5	2	5×10^3	17000	0.0
	4	3×10^5	0	5×10^3	7900	0.0
	5	3 x 10^5	0	5x10^3	21600	0.0
	6	3 x 10^5	0	5x10^3	14600	0.0
**Diluent**	1	3 x 10^5	0	5x10^3	30900	0.0
	2	3 x 10^5	0	5x10^3	7100	0.0

Supplementary Table 4: Treatment and testing delivery of bivalent combination influenza vaccine in mice study

Group	n	mRNA 1	mRNA 2	LNP	mRNA Dose (μg)	Description	Ca09 HAI	Sing16 HAI	Mich15 NAI	Perth09 NAI
1	8	Sing16 H3	Perth09 N2	Yes	0.2, 0.2	co-encapsulated		X		X
2	8	Sing16 H3	Perth09 N2	Yes	0.2, 0.2	separate		X		X
3	8	Ca09 H1	Sing16 H3	Yes	0.2, 0.2	co-encapsulated	X	X		
4	8	Ca09 H1	Sing16 H3	Yes	0.2, 0.2	separate	X	X		
5	8	Mich15 N1	Perth09 N2	Yes	0.2, 0.2	co-encapsulated			X	X
6	8	Mich15 N1	Perth09 N2	Yes	0.2, 0.2	separate			X	X
7	8	Ca09 H1	nc mRNA	Yes	0.2, 0.2	co-encapsulated	X			
8	8	Sing16 H3	nc mRNA	Yes	0.2, 0.2	co-encapsulated		X		
9	8	Perth09 N2	nc mRNA	Yes	0.2, 0.2	co-encapsulated				X
10	8	Mich15 N1	nc mRNA	Yes	0.2, 0.2	co-encapsulated			X	
11	8	Diluent	-	No-	0	single	X	X	X	X

Supplementary Table 5: Statistical analysis for evaluation of bivalent combination influenza mRNA vaccine in mice

**H3H1 Bivalent**<br>H1 comparison at all time point	Fold Difference	95% Lower Confidence Limit	95% Upper Confidence Limit	p-value
Co-encapsulated vs. Separate	x1.5	0.7	3.3	0.2597

**H3H1 Bivalent**<br>H3 group comparison on day 42	Fold Difference	95% Lower Confidence Limit	95% Upper Confidence Limit	p-value
Co-encapsulated vs. Separate	x1.1	0.5	2.6	0.8389

**H3N2 Bivalent**<br>H3 group comparison on day 42	Fold Difference	95% Lower Confidence Limit	95% Upper Confidence Limit	p-value
Co-encapsulated vs. Separate	x1.7	0.9	3.1	0.0904

**H3N2Bivalent**<br>N2 comparison at all time point	Fold Difference	95% Lower Confidence Limit	95% Upper Confidence Limit	p-value
Co-encapsulated vs. Separate	x1.2	0.9	1.6	0.2960

**N1N2 Bivalent**<br>N1 group comparison day 42	Fold Difference	95% Lower Confidence Limit	95% Upper Confidence Limit	p-value
Co-encapsulated vs. Separate	x1.5	1.0	2.1	0.0291

**N1N2 Bivalent**<br>N2 comparison at all time point	Fold Difference	95% Lower Confidence Limit	95% Upper Confidence Limit	p-value
Co-encapsulated vs. Separate	x1.2	0.8	2.0	0.3899

Supplementary Table 6: Treatment and testing delivery of quadrivalent combination influenza mRNA vaccine in NHP

Gr.	n	mRNA 1	mRNA 2	mRNA 3	mRNA 4	LNP	Dose (µg)	Cal09 HAI	Sing16 HAI	Mich15 NAI	Perth09 NAI
1	6	Cal09 H1	Sing16 H3	Mich15 N1	Perth09 N2	YES	10 µg (2.5 µg each), IM Co-encapsulated	X	X	X	X
2	6	nc mRNA	Sing16 H3	nc mRNA	Perth09 N2	YES		X	X	X	X
3	6	Cal09 H1	nc mRNA	Mich15 N1	nc mRNA	YES		X	X	X	X
4	6	Cal09 H1	nc mRNA	nc mRNA	nc mRNA	YES		X	X		
5	6	nc mRNA	Sing16 H3	nc mRNA	nc mRNA	YES		X	X		
6	6	nc mRNA	ncmRNA	Mich15 N1	nc mRNA	YES				X	X
7	6	nc mRNA	nc mRNA	nc mRNA	Perth09 N2	YES				X	X

Supplementary Table 7: Statistical analysis for evaluation of quadrivalent combination influenza mRNA vaccine in NHP

**Comparison of Cal09 HAI in Mono-or Bi- to Quadrivalent formulations (day 42 titers)**		**Fold (95% CI)**	**p-value**
Monovalent<br>Cal09 H1 + 3x nc mRNA	Quadrivalent<br>Cal09 H1 + Sing16 H3 + Mich15 N1 + Perth09 N2	/1.1 (0.4-3.0)	p=0.9054, (NS)
Bivalent<br>Cal09 H1 + Mich15 N1 + 2x nc mRNA		x1.1 (0.4-3.0)	p=0.8002, (NS)

Mixed effect model for repeated measures in the group.

**Comparison of Sing16 HAI in Mono-or Bi- to Quadrivalent formulations (day 42 titers))**		**Fold (95% CI)**	**p-value**
Monovalent<br>Sing16 H3 + 3x nc mRNA	Quadrivalent<br>Cal09 H1 + Sing16 H3 + Mich15 N1 + Perth09 N2	x2.1 (0.6-8.0)	p=0.2501 (NS)
Bivalent<br>Sing16 H3 + Perth09 N2 + 2x nc mRNA		x1.4 (0.4-5.4)	p=0.5894 (NS)

Mixed effect model for repeated measures with the group, time and their interaction as fixed factor. Time was included as repeated measures.

**Mich15 NAI:** On D42: The descriptive conclusions were that Quadrivalent, bivalent (Cal09 H1+ Mich15 N1 + 2x nc mRNA) and monovalent (Mich15 N1 + 3x nc mRNA) were not different and showed a mean titer >5120

**Comparison of Perth09 in Mono-or Bi- to Quadrivalent formulations (day 42 titers)**		**p-value**
Monovalent<br>Perth09 N2 + 3x nc mRNA	Quadrivalent<br>Cal09 H1 +<br>Sing16 H3 +<br>Mich15 N1 +<br>Perth09 N2	p=0.8485 (NS)
Bivalent<br>Sing16 H3 + Perth09 N2 + 2x nc mRNA		p=0.4545 (NS)
